# Characterization of Three Novel IMP Metallo-β-Lactamases, IMP-89, IMP-91, and IMP-96, and Diverse *bla*_IMP_-Carrying Accessory Genetic Elements from Chinese Clinical Isolates

**DOI:** 10.1128/spectrum.04986-22

**Published:** 2023-04-19

**Authors:** Xinyue Li, Xiaofei Mu, Fangzhou Chen, Xiuhui Lu, Jiaqi He, Yali Zheng, Dongsheng Zhou, Zhe Yin, Peng Wang

**Affiliations:** a State Key Laboratory of Pathogen and Biosecurity, Beijing Institute of Microbiology and Epidemiology, Beijing, China; Universitat Greifswald

**Keywords:** antimicrobial resistance, IMP-type MBLs, accessory genetic elements, genome sequencing, comparative genomic analysis

## Abstract

Three novel imipenemase (IMP)-type metallo-β-lactamases (MBLs), referred to as IMP-89, IMP-91, and IMP-96, were detected in three clinical isolates from China. Antimicrobial susceptibility tests indicated these novel enzymes were resistant to most β-lactams, and IMP-96 with a Ser262Gly mutation had higher activity against meropenem than its point mutant. We then collected sequence data on all 91 available IMP variants for phylogenetic analysis. To further analyze the genetic environment of *bla*_IMP_, an extensive comparison was applied to nine accessory genetic elements (AGEs), including six sequenced *bla*_IMP_-carrying AGEs in this study and three others from GenBank. These nine AGEs were divided into three groups: three Inc_pJBCL41_ plasmids, Tn*6417* and its two derivatives, and three Tn*6879*-related integrative and conjugative elements (ICEs). All *bla*_IMP_ genes in this study were captured by class 1 integrons. In the integrons, *bla*_IMP_ genes usually coexisted with other resistance genes, which further impeded clinical antibacterial treatment. The emergence of new IMP variants and the diversity and complexity of their genetic environment make the prevention and control of drug-resistant strains critical, requiring serious attention from clinical and public health management departments.

**IMPORTANCE** The spread of IMP-type MBLs has increased dramatically in recent years. We discovered three novel IMP variants from three clinical isolates in China. We summarized the classification and evolutionary relationship of all available IMP variants. Moreover, we detailed the genetic characteristics of *bla*_IMP_-carrying accessory genetic elements in five clinical isolates. Given the risk of rapid and extensive spread of *bla*_IMP_ genes, we suggest that continuous surveillance is crucial to combat the acquisition and transmission of *bla*_IMP_ genes by bacteria, which can impede clinical therapy effectiveness.

## INTRODUCTION

Imipenemases (IMPs), one of the most common metallo-β-lactamase (MBL) in clinical isolates (1), are enzymes able to hydrolyze nearly all β-lactams (2), and specifically, the carbapenems that are last-resort antibiotics against various multidrug-resistant bacterial infections (3). Currently, at least 91 IMP variants have been discovered worldwide (https://www.ncbi.nlm.nih.gov/pathogens/refgene), and extensive spread of IMPs has been demonstrated in Japan and China (4). Full-length IMPs typically consist of 246 amino acid residues, and amino acid substitutions occurring at different positions alter an IMP’s conformation, resulting in a diverse array of IMPs with variable hydrolytic activity toward different β-lactam antibiotics.

The *bla*_IMP_ carbapenemase genes encoding diverse IMP variants are frequently captured by class 1 integrons (5) that are embedded in accessory genetic elements (AGEs), allowing horizontal transfer and widespread movement of *bla*_IMP_. Researchers have noted the spread of *bla*_IMP_ to various plasmids in different incompatibility (Inc) groups such as IncN (6), IncL/M (7), IncHI2 (8), IncF (9), Inc_pRBL16_ (10), and IncHI5 (11). ICEs can transfer from one cell to another cell through self-integration and self-conjugation modules (12). IMEs are not self-transmissible but achieve intercellular mobility with the help of other conjugative elements containing conjugal transfer genes (13). A unit transposon carries a core transposition module to achieve horizontal gene transfer (14). Our previous studies (15–17) have shown genetic characteristics of novel transposons carrying *bla*_IMP_ (*bla*_IMP-8_-carrying ICE Tn*6397*, *bla*_IMP-38_-carrying unit transposon Tn*6382*, and *bla*_IMP-1_-carrying unit transposon Tn*6910*).

In this study, five IMP-producing clinical isolates from China were fully sequenced. Three novel IMP variants, namely IMP-89, IMP-91, and IMP-96, were detected. The three enzymes were viable and conferred resistance to nearly all antibiotics tested. IMP-96 showed an increased MIC value specifically toward meropenem in Escherichia coli because of its critical Ser262Gly substitution. A genomic phylogenetic analysis of 91 available IMP variants suggested the three novel IMP variants belong to three different phylogenetic groups. We further performed a genetic analysis of nine AGEs, including six *bla*_IMP_-carrying AGEs sequenced from the five clinical isolates in this study and three prototype AGEs from GenBank. Thus, this study identifies three novel IMP variants and provides a deeper understanding of the genetic diversification of IMP-encoding AGEs, highlighting the emergence and spread of the resistance genes *bla*_IMP_.

## RESULTS

### Cloning and heterologous expression of *bla*_IMP-89_, *bla*_IMP-91_, and *bla*_IMP-96_.

**FIG 1:**
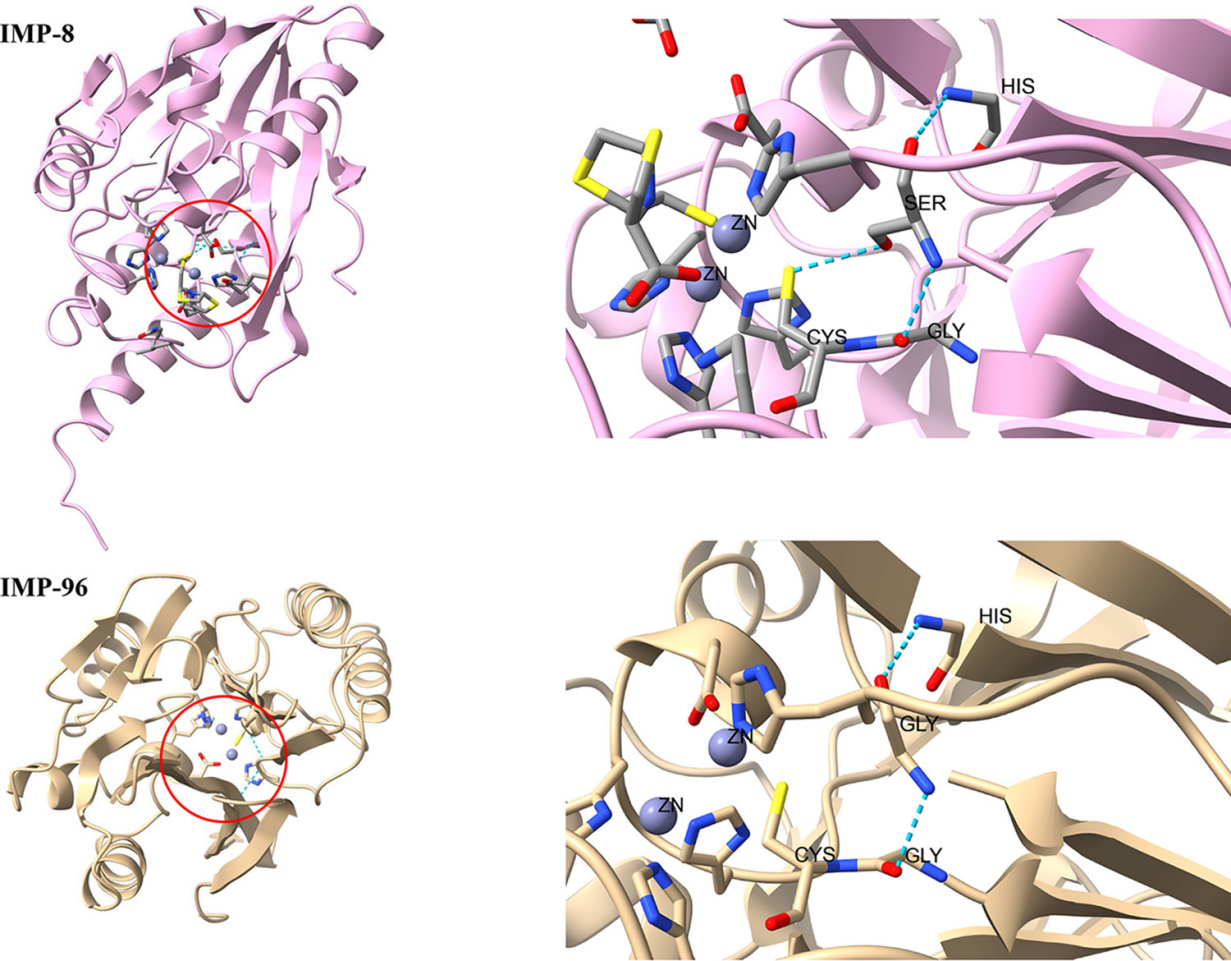
Ser262Gly mutation of IMP-96. Structural features of IMP-8 and IMP-96. The hydrogen bonds between residue 262 with other amino acid residues are highlighted with a red circle. The image was created using ChimeraX (https://www.rbvi.ucsf.edu/chimerax).

The complete genome of each of the five IMP-producing clinical isolates from China was sequenced (Table S1 in the supplemental material). Three novel IMP variants, namely IMP-89, IMP-91, and IMP-96 (Fig. S1), were detected from three different clinical isolates: Pseudomonas putida NY5709, P. aeruginosa NY3045, and *Stenotrophomonas* spp. NY11291, respectively. The open reading frame of the three novel variants IMP-89/91/96, their point mutants IMP-26/14/8, and the representative IMP variant IMP-1 (used as a positive control) were cloned into a kanamycin-resistant pUC57 vector and then transformed into E. coli TOP10 to obtain the seven corresponding E. coli electroporants, pUC57K-*bla*_IMP-89/26/91/14/96/8/1_-TOP10. Strains encoding IMP-89, IMP-91, and IMP-96 enzymes were active and had resistance to cephem (cefazolin, ceftazidime, and cefoxitin) and carbapenem (meropenem) relative to the negative-control strains TOP10, TOP10/pUC57K, and ATCC 25922 but remained susceptible to aztreonam. IMP-89 and IMP-96 were also resistant to carbapenem (imipenem) but remained susceptible to penam (ampicillin). In contrast, IMP-91 could hydrolyze ampicillin but was susceptible to imipenem. The hydrolytic activity of IMP-89/91 was relatively poor against the antibiotics tested compared with IMP-26/IMP-14. However, the E. coli TOP10 expressing IMP-96 had increased resistance toward meropenem with a higher MIC value than the strain expressing *bla*_IMP-8_ (Table 1). Molecular representation of the structure of IMP-8 and IMP-96 (Fig. 1) indicated that the Ser262Gly mutation disrupted a hydrogen-bonding interaction with the catalytic residue Cys221, resulting in increased resistance to meropenem.

**TABLE 1:** MICs of selected antimicrobial agents for the transformants carrying *bla*_IMP_

Bacterial isolate	Minimum inhibitory concemtration (μg/mL)/antimicrobial susceptibility						
	Cefazolin	Ceftazidime	Cefoxitin	Meropenem	Imipenem	Ampicillin	Aztreonam
TOP10/pUC57K-IMP-1	256	16	128	32	4	16	≤1
TOP10/pUC57K-IMP-89	512	128	128	32	8	16	≤1
TOP10/pUC57K-IMP-26	>512	>512	256	128	32	64	≤1
TOP10/pUC57K-IMP-91	512	128	128	64	≤2	32	≤1
TOP10/pUC57K-IMP-14	>512	>512	>512	256	256	>512	≤1
TOP10/pUC57K-IMP-96	512	128	512	128	8	16	≤1
TOP10/pUC57K-IMP-8	>512	>512	>512	64	>512	512	≤1
TOP10/pUC57K	≤4	0.25	8	≤0.25	≤0.25	8	≤1
TOP10	≤4	0.25	≤4	≤0.25	≤0.25	8	≤1
ATCC 25922	≤4	0.25	≤4	≤0.25	≤0.25	8	≤1

### Evolution and classification of IMP-type MBLs.

**FIG 2:**
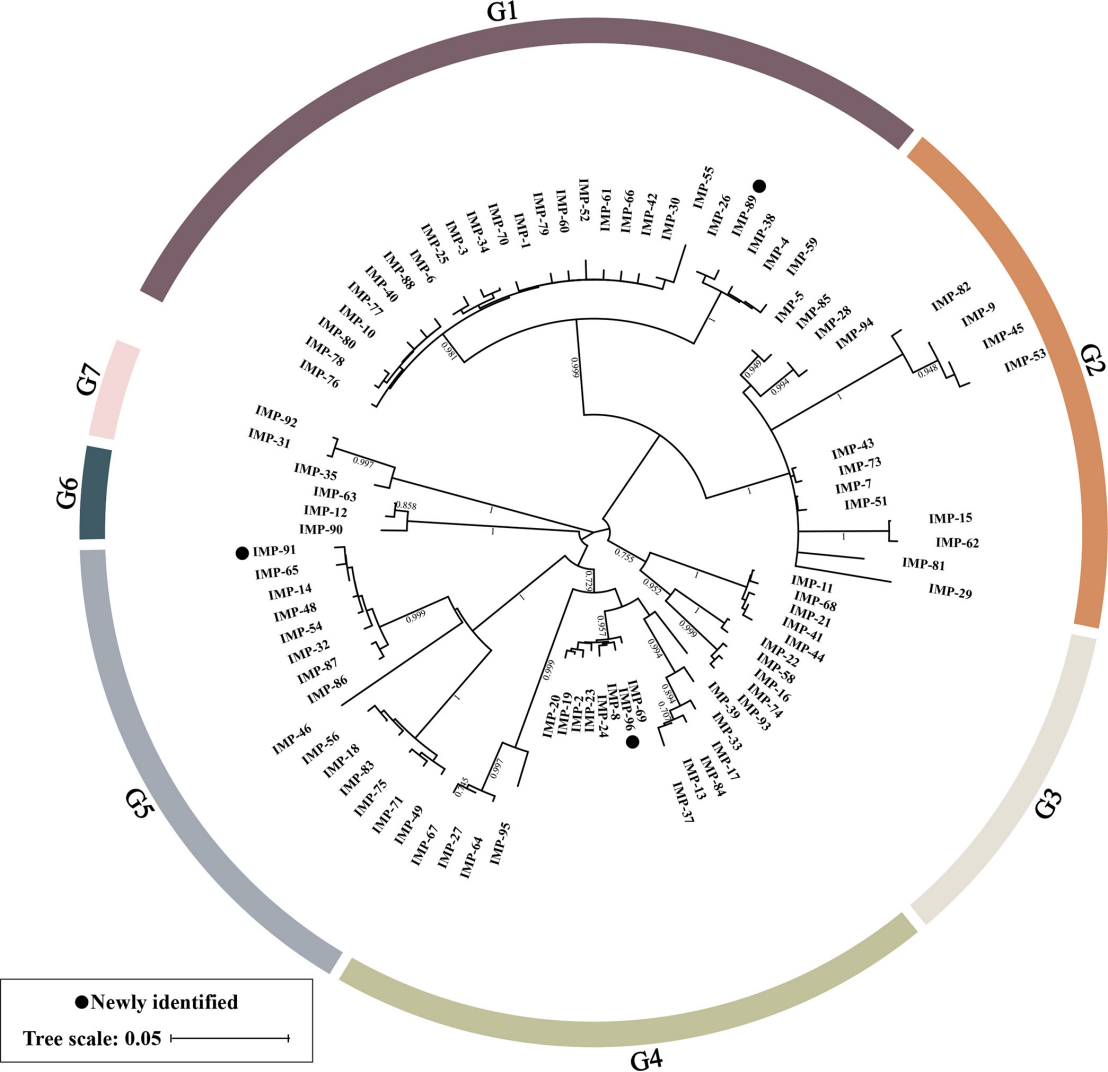
Phylogenetic tree of the 91 IMP variants. Circles denote the three novel IMP variants identified in this study, other variants are from the GenBank database. The degree of support (percentage) for each cluster of associated taxa, as determined by bootstrap analysis, is shown next to each branch. Bar corresponds to scale of sequence divergence.

A phylogenetic tree (Fig. 2) was constructed based on the amino acid sequences of all 91 available IMP variants (Table S2), including the three novel IMP variants identified in this study. All IMP variants could be assigned to seven groups (G1 to G7), with significant phylogenetic distance between each group. Classification results were also verified by pairwise comparison of the amino acid sequences of IMPs (Fig. S2 and Table S3), which showed that the genetic identity of IMPs in each group was generally ≥90%. IMP-89, IMP-91, and IMP-96 belonged to groups G1, G5, and G4, respectively. We further considered the countries where these IMP variants first appeared (Fig. S3). The endemic spread of IMP-type enzymes had been reported in Japan and China (18). Overall, these data suggested complex genetic mutations and spread of IMP-type MBLs.

### A collection of nine AGEs for detailed sequence comparison.

We identified two *bla*_IMP_-carrying plasmids and four *bla*_IMP_-carrying chromosomal AGEs from the five isolates we collected. A detailed sequence comparison analysis applied to these six AGEs together with three prototype AGEs from GenBank (Table 2) revealed that these AGEs are composed of three distinct groups: (i) three Inc_pJBCL41_ plasmids pJBCL41, pNY5709-IMP, and pNY11382-IMP; (ii) three related ICEs Tn*6879*, Tn*6880*, and Tn*6881*; and (iii) Two Tn*6417* derivatives Tn*7450* and T6417RE_NY3045_-2. Elements T6417RE, T1403RE, and T6483RE (see below) could not be recognized as intact transposons due to the truncation of relevant core transposition modules.

**TABLE 2:** Major features of AGEs characterized in this study

Group	AGE	Accession no.	Nucleotide position	Length (bp)	*bla*_IMP_	*bla*_IMP_-carrying integron	Host bacterium	Reference
Inc_pJBCL41_	pJBCL41	MK496050		498,516			Pseudomonas *sp.* FFUP_PS_41	19
	pNY5709-IMP	MN961670		636,818	*bla*_IMP-89_	In1783	Pseudomonas putida NY5709	This study
	pNY11382-IMP	CP097104		480,951	*bla*_IMP-34_	In2089	Pseudomonas *sp.* NY11382	This study
Tn*6417*-related elements	Tn*6417*	CP013993	Chromosome (5365108.0.5473293)	108,186			Pseudomonas aeruginosa DHS01	22
	Tn*7450*	CP096975	Chromosome (3808714.0.3890907)	82,194	*bla*_IMP-96_	In2092	*Stenotrophomonas sp.* NY11291	This study
	T6417RE_NY3045_-2	CP059995	Chromosome (5270031.0.5293475)	23,445	*bla*_IMP-91_	In1792	Pseudomonas aeruginosa NY3045	This study
Tn*6879*-related ICEs	Tn*6879*	CP033104	Chromosome (1586424.0.1680337)	93,914			Pseudomonas *sp.* LTGT-11-2Z	25
	Tn*6880*	CP045554	Chromosome (3880617.0.3970693)	90,077	*bla*_IMP-1_	In1771	Pseudomonas *sp.* NY5710	This study
	Tn*6881*	CP045551	Chromosome (1445820.0.1535851)	90,032	*bla*_IMP-1_	In1768	Pseudomonas putida NY5709	This study

### Genetic environment of *bla*_IMP-89_ in NY5709.

*bla*_IMP-89_ was identified in pNY5709-IMP from the P. putida isolate NY5709. A *bla*_IMP-34_-carrying plasmid pNY11382-IMP was found from the Pseudomonas isolate NY11382. These two plasmids (Fig. S4) were identified as belonging to the Inc_pJBCL41_ group because they had identical *repA* genes sharing ≥95% nucleotide identity to *repA*_IncpJBCL41_. Found in *P. shirazica* FFUP_PS_41, pJBCL41was reported to be a 498,516-bp megaplasmid with limited similarity to publicly identified plasmids (19), therefore, was proposed to be a novel incompatibility group, Inc_pJBCL41_. pJBCL41 represented the Inc_pJBCL41_ reference plasmid, because it had the most complete Inc_pJBCL41_ backbone with the least exogenous insertions. All Inc_pJBCL41_ plasmids from public databases were megaplasmids, with lengths >490 kb. The complete nucleotide sequence of pNY5709-IMP was 636,818 bp in length. To the best of our knowledge, pNY5709-IMP is the largest multidrug resistance plasmid reported to date in Pseudomonas.

The three plasmids had >90% nucleotide identity across >85% of their backbone sequences, which contained the key genes of Inc_pJBCL41_-type plasmids, including *repA*_IncpJBCL41_ (replication), *parAB*-*parB2* (partition), *che* (chemotaxis), *ter* (tellurite resistance protein), *umuCD* (DNA polymerase V protein), *cpl* (coupling protein), and several *tivF* genes (conjugal transfer). Several differences in the backbones of the three plasmids were observed: (i) pJBCL41 harbored its four unique backbone regions *orf879*-to-*orf855*, *orf303*-to-*orf948*, *orf1005*-to-*orf1125*, and *orf141*-to-*orf810*; (ii) *orf1203*-to-*araC*, *orf987*-to-*orf411*, and *orf450*-to-*orf1290* regions were found only in pNY5709-IMP; and (iii) pNY11382-IMP had its unique *orf303*-to-*orf480* region, and was missing the *orf252*-to-*orf633* region. Two large-fragment inversions of the backbone region were also observed on pNY11382-IMP.

**FIG 3:**
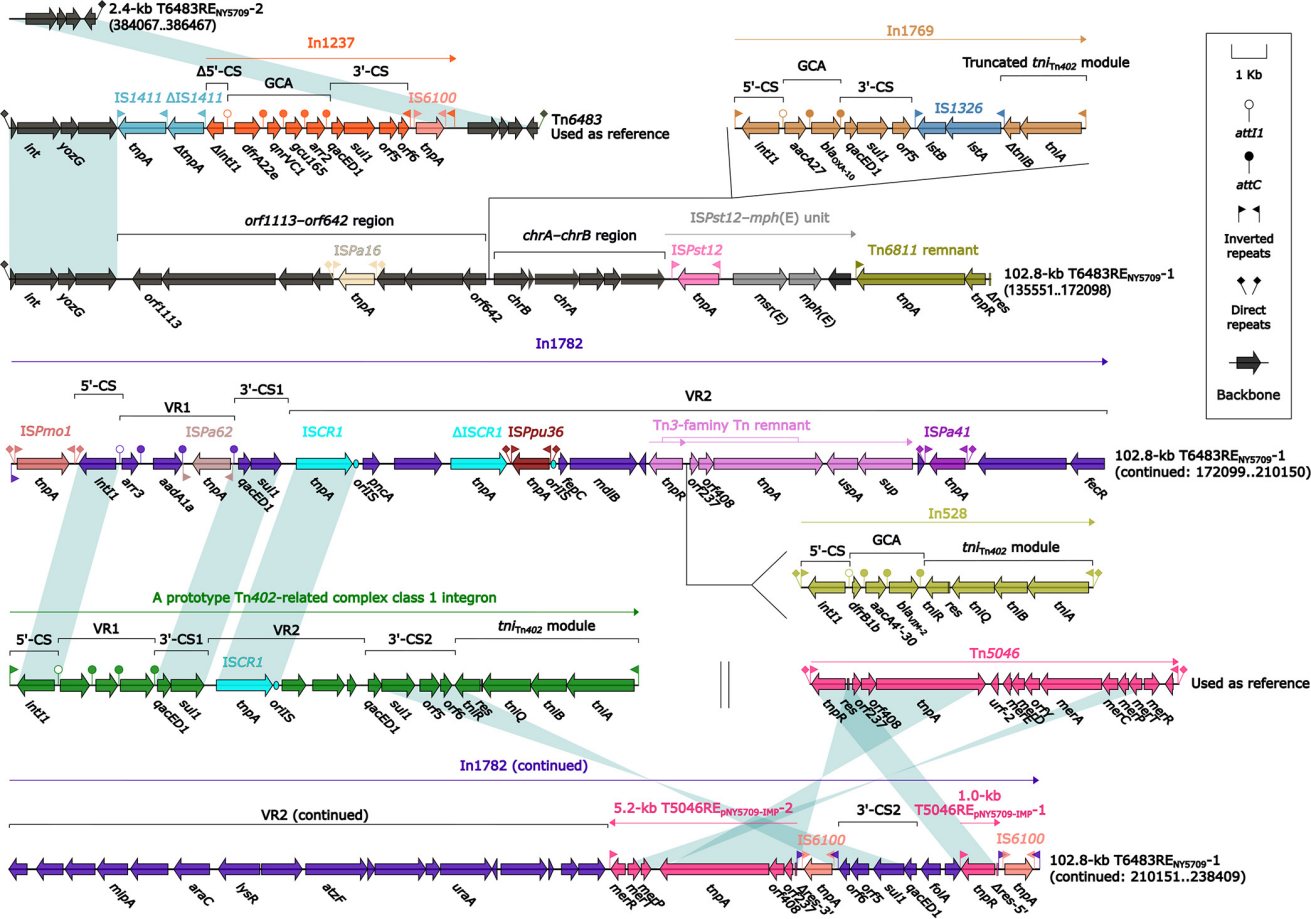
Organization of accessory modules from pNY5709-IMP. Genes are denoted by arrows. Genes, AGEs, and other features are colored based on their functional classification. Shading denotes regions of homology (nucleotide identity ≥90%). Numbers in brackets indicate nucleotide positions within the corresponding plasmid. The accession numbers of Tn*6483* and Tn*5046* used as references are KY883660 and MF344568, respectively.

**FIG 4:**
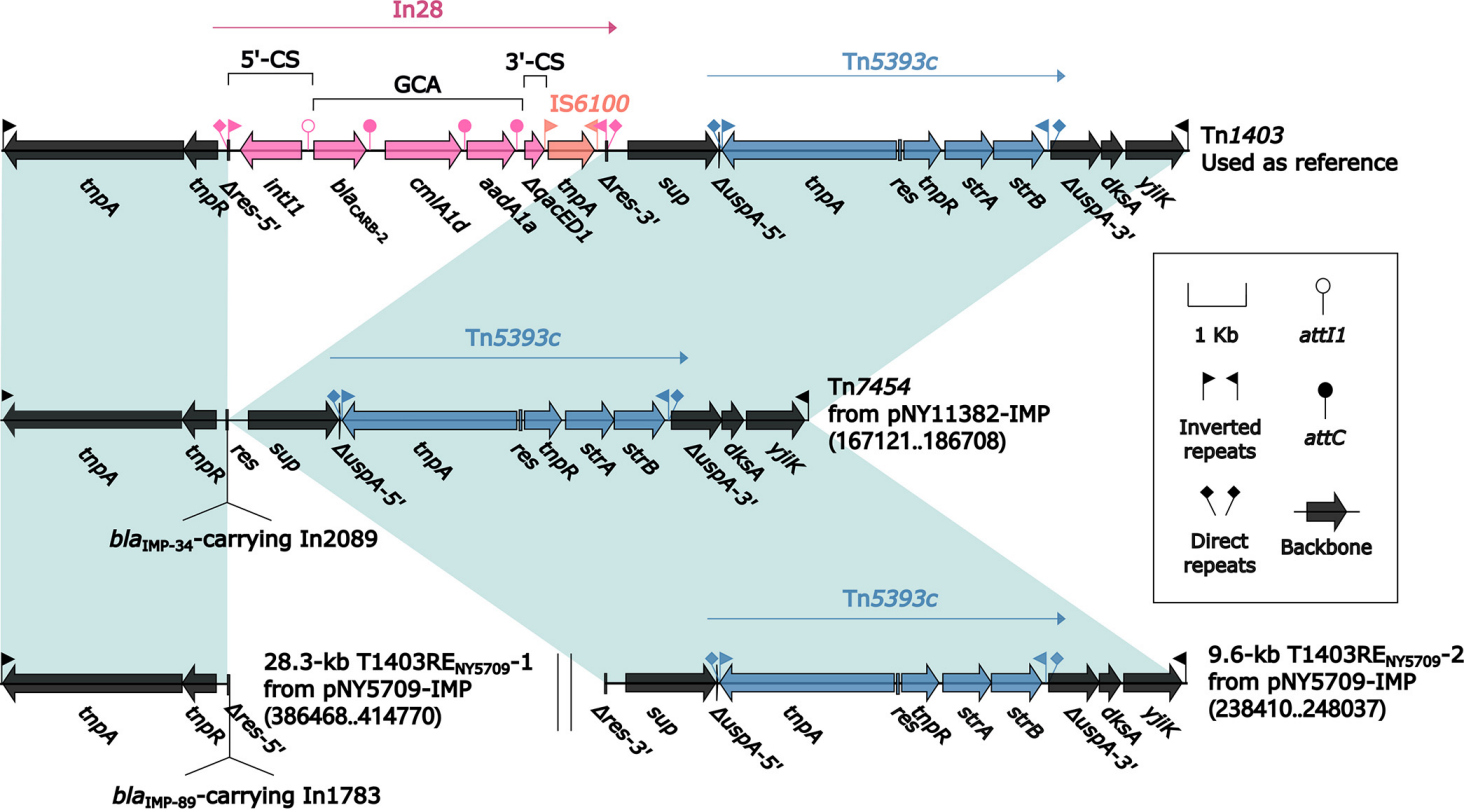
Comparison of Tn*1403*-related elements. Genes are denoted by arrows. Genes, AGEs, and other features are colored based on their functional classification. Shading denotes regions of homology (nucleotide identity ≥90%). Numbers in brackets indicate nucleotide positions within the plasmids of strains NY11382 and NY5709.

Many different accessory modules were identified at various sites within the backbones of these three plasmids. In addition to a large number of IS elements inserted into these plasmids, each of the three plasmids carried at least one major accessory module. Tn*7453* from pJBCL41 has been described in detail (19). 102.8-kb T6483RE_NY5709_-1 and 2.4-kb T6483RE_NY5709_-2 were found in pNY5709-IMP; these were identified as derivatives of Tn*6483*. The prototype IME Tn*6483* was initially found in P. putida SY153 (accession number KY883660). T6483RE_NY5709_-1 and Tn*6483* (Fig. 3) shared the core IME backbone markers *int* (integrase) and *attL* (attachment sites at the left end) but exhibited three major modular variations: (i) T6483RE_NY5709_-1 contained the unique backbone regions *orf1113*-to-*orf642* and *chrA*-*chrB*; (ii) Tn*6483* harbored only one antimicrobial resistance locus (ARL), a concise class 1 integron, In1237; and (iii) T6483RE_NY5709_-1 included a concise class 1 integron In1769 with the gene cassette array (GCA) *aacA27*–*bla*_OXA-10_, an IS*Pst12*-*mph*(E) unit, and a 3.7-kb Tn*6811* remnant together with In1782. In1782 was a complex class 1 integron that harbored two variable regions: GCA/VR1 (*arr3*–*aadA1a*) and VR2. A concise class 1 integron, In528, and T5046RE_NY5709_ were inserted into the VR2 of In1782. In528 harbored GCA *dfrB1b*-*aacA4'-30*-*bla*_VIM-2_, interrupting a Tn*3*-family Tn remnant. A 3′-CS2 inversion was found in In1782, resulting from the insertion of IS*6100* into the *res* (resolution site) site of T5046RE_NY5709_ and leading to the disruption of T5046RE_NY5709_. The 2.4-kb T6483RE_NY5709_-2 shared the remaining backbone region with Tn*6483*. Thus, pNY5709-IMP was a multidrug-resistant plasmid that carried multiple β-lactams genes, including *bla*_IMP-89_, *bla*_VIM-2_, and *bla*_OXA-10_. In1769 (Fig. S5), found in pNY11382-IMP, was a concise class 1 integron with GCA *aacA27*–*bla*_OXA-10_ and acquired a 4.5-kb Tn*6758* remnant, a 12.2-kb Tn*6855* remnant, and ΔTn*5393c*. In addition, three Tn*1403* derivatives, Tn*7454*, T1403RE_NY5709_-1, and T1403RE_NY5709_-2, were found in pNY11382-IMP and pNY5709-IMP (Fig. 4).

Tn*1403* was a Tn*3*-family prototype unit transposon, originally found in P. aeruginosa plasmid RPL11 (20) and composed of the core backbone structure IRL (inverted repeat left)-*tnpA* (transposase)-*tnpR* (resolvase)-*res*-*sup*-*uspA*-*dksA*-*yjiK*-IRR (inverted repeat right), with a concise class 1 integron, In28, inserted into *res* and Tn*5393c* inserted into *uspA* (21). Tn*7454* and T1403RE_NY5709_-1 differed from Tn*1403* by acquisition of a *bla*_IMP-34_-carrying concise class 1 integron In2089 and a *bla*_IMP-89_-carrying complex class 1 integron In1783 (see below), respectively, instead of In28.

### Genetic environment of *bla*_IMP-91_ in NY3045 and *bla*_IMP-96_ in NY11291.

**FIG 5:**
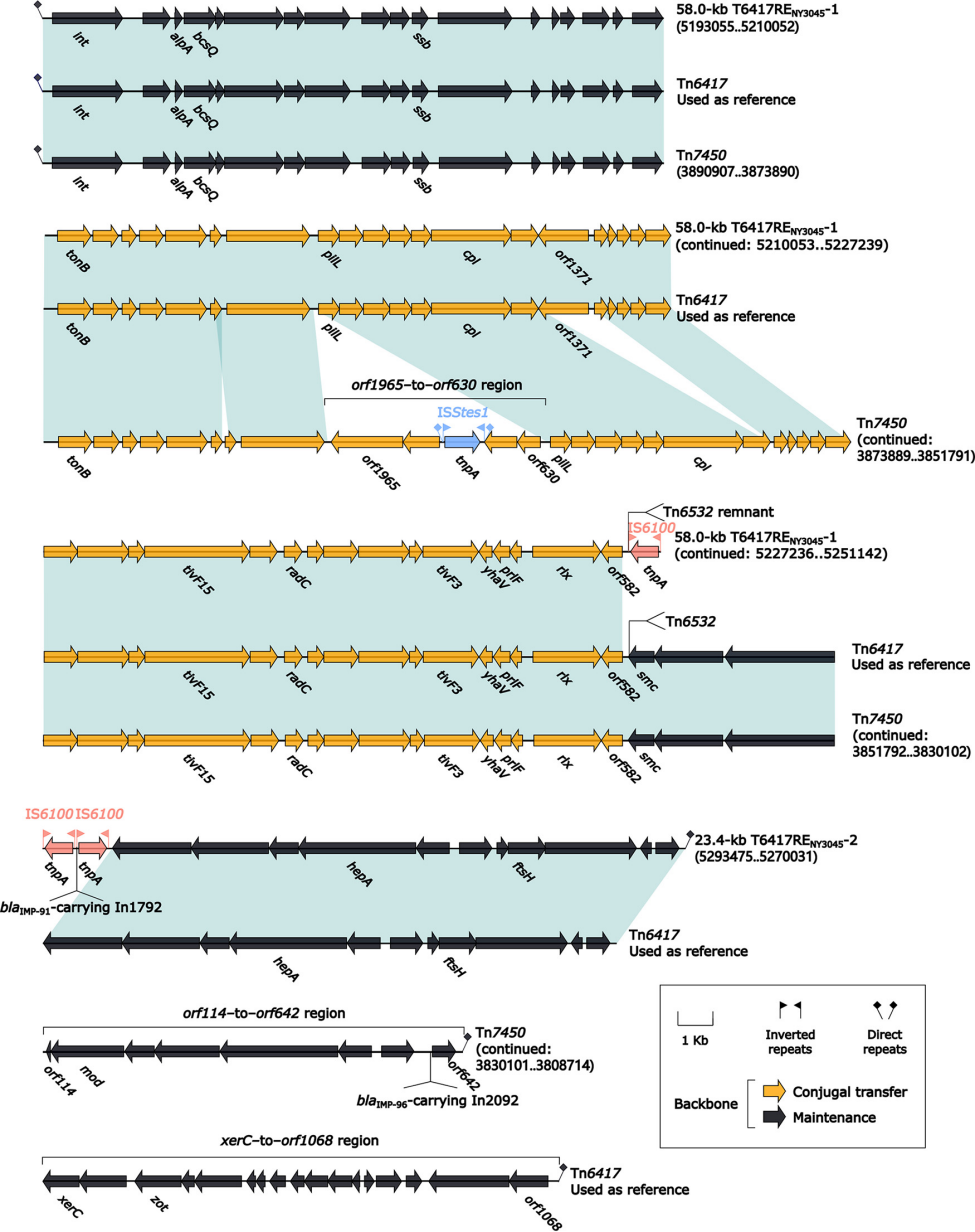
Comparison of three Tn*6417*-related elements. Genes are denoted by arrows. Genes, AGEs, and other features are colored based on their functional classification. Shading denotes regions of homology (nucleotide identity ≥90%). Numbers in brackets indicate nucleotide positions within the chromosomes of strains NY3045 and NY11291.

We did a detailed sequence comparison of Tn*6417* and three of its derivatives: *bla*_IMP-96_-carrying Tn*7450* detected in the *Stenotrophomonas* strain NY11291 and *bla*_IMP-91_-carrying T6417RE_NY3045_-2 and T6417RE_NY3045_-1 (found upstream of T6417RE_NY3045_-2) (Fig. 5) from the P. aeruginosa strain NY3045. The prototype ICE Tn*6417* was initially described in P. aeruginosa DHS01 (22) and contained a core backbone structure of *attL/R*, *int*, *cpl*, *rlx* (relaxase), and an F-type T4SS gene set. The three Tn*6417* derivatives were inserted into three different chromosomal locations and exhibited modular variations across their backbones: (i) *xerC*-to-*orf1068* region from Tn*6417* was absent in all three derivatives; (ii) Tn*7450* had its unique *orf1965*-to-*orf630* and *orf114*-to-*orf642* regions; and (iii) only T6417RE_NY3045_-1 contained *orf1371*. Each of these derivatives carried different main accessory modules: a *bla*_IMP-96_-carrying concise class 1 integron In2092 (see below) in Tn*7450*, a 7.0-kb Tn*6532* remnant in T6417RE_NY3045_-1, and a *bla*_IMP-91_-carrying concise class 1 integron In1792 (see below) in T6417RE_NY3045_-2.

No therapeutic options exist to treat *Stenotrophomonas* infections with β-lactams. Based on *in vitro* activity, the “drug of choice” for *Stenotrophomonas* has long been trimethoprim-sulfamethoxazole (SXT) (23); however, our MIC experiments (Table S1) showed that NY11291 had high-level resistance to SXT (MIC ≥ 320). Tn*7450* from NY11291 harbored the *sul1* gene at the 3′ end of the concise class 1 integron In212 (see below). The *sul1* genes have been reported to associate with the class 1 integrons and contribute to the resistance to SXT (24).

### Three related ICEs Tn*6879*, Tn*6880*, and Tn*6881*.

**FIG 6:**
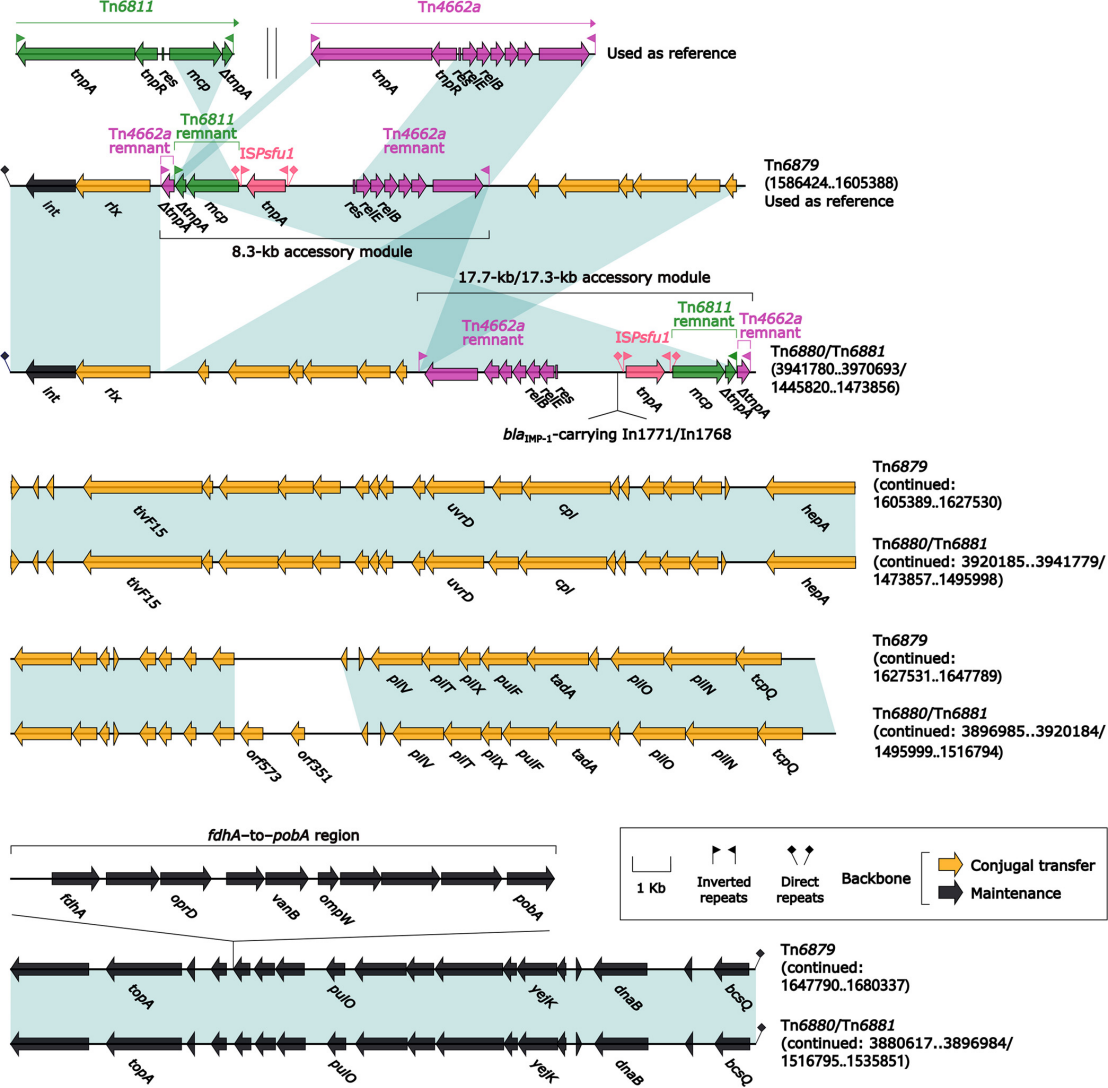
Comparison of three related ICEs Tn*6879*, Tn*6880*, and Tn*6881*. Genes are denoted by arrows. Genes, AGEs, and other features are colored based on their functional classification. Shading denotes regions of homology (nucleotide identity ≥90%). Numbers in brackets indicate nucleotide positions within the chromosomes of strains LTGT-11-2Z, NY5710, and NY5709, respectively. The accession numbers of Tn*6811* and Tn*4662a* (41) used as references were CP029148 and KJ920396, respectively.

Whole-genome sequencing of strain NY5709 confirmed the presence of another *bla*_IMP_ gene in a chromosome-borne AGE Tn*6881*. We also detected *bla*_IMP-1_-carrying Tn*6880* in the Pseudomonas strain NY5710. Then, a detailed sequence comparison was applied to Tn*6880*, Tn*6881*, and the prototype ICE Tn*6879*. Tn*6879* was initially found in Pseudomonas spp. LTGT-11-2Z (25). All three ICEs were integrated 92-bp upstream of the chromosomal gene *queC* (queuosine biosynthesis protein) and shared the core backbone markers *attL*/*R*, *int*, *rlx*, and *cpl* (Fig. 6). However, the backbone of Tn*6879* had a unique *fdhA*-to-*pobA* region; correspondingly, Tn*6880* and Tn*6881* carried their unique *orf573* and *orf351*, respectively. Each of the three ICEs carried a single accessory module integrated at the same site. Tn*6879* had no resistance genes, while Tn*6880* and Tn*6881* harbored *bla*_IMP-1_-carrying concise class 1 integrons In1771 and In1768, respectively (see below).

### Comparison of six *bla*_IMP_-carrying integrons.

**FIG 7:**
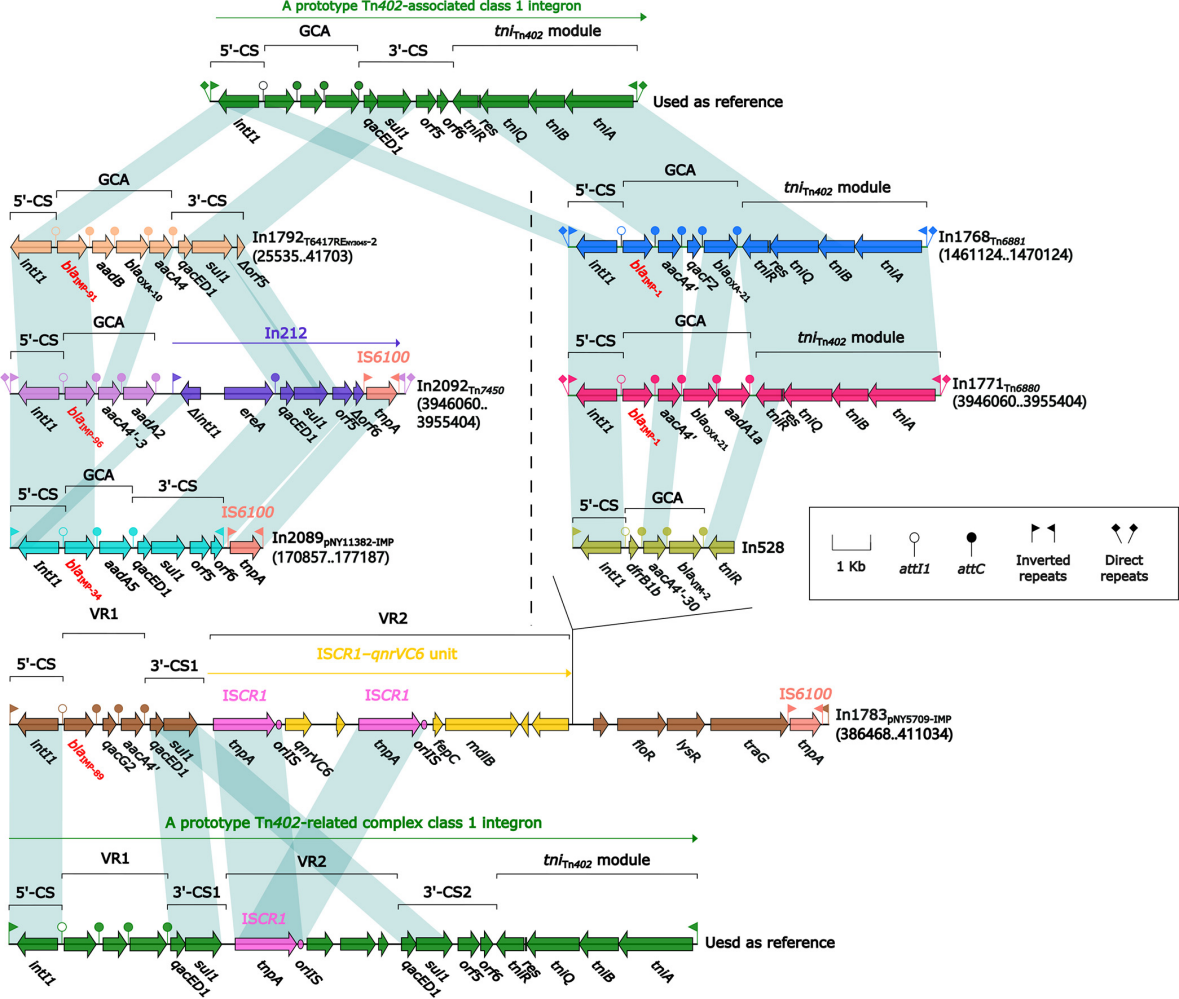
Comparison of the six *bla*_IMP_-carrying class 1 integrons. Genes are denoted by arrows. Genes, AGEs, and other features are colored based on their functional classification. Shading denotes regions of homology (nucleotide identity ≥90%). Numbers in brackets indicate nucleotide positions within the chromosomes or plasmids of strains NY3045, NY11291, NY11382, NY5709, and NY5710.

A detailed genetic dissection analysis was applied to six *bla*_IMP_-carrying integrons found in the five fully sequenced isolates (Fig. 7), containing five *bla*_IMP_ genotypes: *bla*_IMP-1_, *bla*_IMP-34_, *bla*_IMP-89_, *bla*_IMP-91_, and *bla*_IMP-96_. The basic integron platform consists of the following: *intI* (integrase), Pc (promoter), *attI* (recombination site), GCA, and *attC* (recognition site). Five of these were concise class 1 integrons. In1792, In2089, In1768, and In1771 contained four different GCAs as their sole ARLs, and In2092 contained *bla*_IMP-96_-*aacA4'-3*-*aadA2* (GCA) and In212 (GCA: *ereA*). One *bla*_IMP_-carrying complex class 1 integron carried three ARLs. In1783 carried *bla*_IMP-89_-*qacG2*-*aacA4'* (VR1), IS*CR1*-*qnrVC6* unit (VR2), and In528 (GCA: *dfrB1b*-*aacA4'-30*-*bla*_VIM-2_).

### Newly identified or designated AGEs.

There were eight newly identified chromosomal AGEs: (i) three were directly integrated into the chromosomes and included the three ICEs Tn*6880*, Tn*6881*, and Tn*7450*; and (ii) the remaining five were the inner components of the above elements directly inserted, including the four integrons In1771, In1768, In1792, and In2092, and the one IS element IS*Stes1*. There were seven newly identified AGEs from plasmids, including the one unit transposon Tn*7454*, the four integrons In1769, In1782, In1783, In2089, and the two IS elements IS*Ppu36* and IS*Psp18*. Additionally, three AGEs were newly designated (first designated in this study, but with previously determined sequences): one ICE Tn*6879*, one unit transposon Tn*7453*, and one IS element IS*Psp17*.

## DISCUSSION

IMP-type enzymes are the first acquired MBLs in Gram-negative bacilli (26). The number of reports on the characterization of novel IMP in various bacterial species has increased worldwide (27). Here, we report on three novel variants of IMP named IMP-89, IMP-91, and IMP-96. These novel IMP variants can hydrolyze nearly all tested antibiotics. IMP-89 and IMP-91 are less resistant than their point mutations IMP-26 and IMP-14, respectively. Notably, the amino acid sequence of IMP-96 differs from its point mutation IMP-8 by the amino acid substitution S262G, resulting in increased resistance against meropenem. The substitution S262G may play an important role in increasing resistance against certain carbapenems in E. coli, as the substitution disrupts a hydrogen-bonding interaction with the base of the L3 loop, altering its conformation (28). This substitution may have arisen due to the selective pressure caused by the use of carbapenem.

To date, 91 IMP variants have been reported worldwide. We first constructed a phylogenetic tree containing the three new variants. Based on the phylogenetic tree and the pairwise comparison of amino acid sequences, IMPs fall into seven groups, and IMP-89, IMP-91, and IMP-96 phylogenetically belong to the groups of G1, G5, and G6, respectively. IMPs are clearly not monophyletic with 1 to 22% sequence dissimilarity. Novel IMP variants continue to be identified in Japan (*n* = 23) and China (*n* = 13). One report indicates increased use of certain carbapenems in Japan in recent years (29), and China has a high rate of antibiotic use for hospital inpatients (30). The rapid evolution and observed spread of IMP highlight a growing threat that needs to be taken seriously.

Few existing reports show diverse *bla*_IMP_-carrying AGEs or provide a systematic summary for them. This study presents the full sequences of six AGEs carrying *bla*_IMP_ genes, including two plasmids and four chromosomal AGEs that can be divided into three distinct groups. The Inc_pJBCL41_ is a novel incompatibility group. The four chromosomal AGEs belong to two different categories: two Tn*6879*-related ICEs Tn*6880* and Tn*6881*; two Tn*6417*-related derivatives Tn*7450* and T6417RE_NY3045_-2. Our results indicate a wide dissemination of *bla*_IMP_ by AGEs, which may become common vehicles mediating the spread of antimicrobial resistance.

Many resistance genes in addition to *bla*_IMP_ coexist in these AGEs. The results of the antimicrobial susceptibility test (Table S1) show that all five strains remain highly drug-resistant. *bla*_IMP_ genes usually coexist in the integrons with other resistance genes, such as the aminoglycoside resistance genes *aacA4* and *aad1*, class D β-lactamase gene *bla*_OXA_, and phenicol resistance gene *catB3*, resulting in multidrug resistance that increases the difficulty of finding a clinical antibacterial treatment. In the conserved region of class 1 integrons, the *sul1* gene also contributes to the problem of drug resistance. *Stenotrophomonas* is typically treated with SXT (31). The harboring of the *sul1* gene in the *bla*_IMP-96_-carrying integron In2092 is the key to the increase of SXT resistance in the *Stenotrophomonas* strain NY11291. Clinical class 1 integrons play a crucial role in spreading antibiotic resistance among bacterial pathogens, and further investigations are needed.

The combination of two MBLs encoded by the *bla*_IMP_ gene and another gene in one strain is becoming more common. Notably, we report the largest multidrug resistance plasmid ever sequenced in the Pseudomonas genus, pNY5709-IMP, which encodes two MBLs, IMP-89, and VIM-2. pNY5709-IMP belongs to Inc_pJBCL41_ plasmids and carries substantial numbers of diverse accessory modules.

In conclusion, five IMP-producing clinical strains were collected from Chinese hospitals, and these strains showed high resistance to various broad-spectrum β-lactams. Three novel IMP variants, IMP-89, IMP-91, and IMP-96 were detected. The presence of S262G in IMP-96 leads to increased resistance against the carbapenem meropenem. Rational and effective clinical use of antibiotics requires investigating the diversity of hydrolysis capacities in IMPs. A phylogenetic reconstruction using all available *bla*_IMP_ protein sequences shows the evolutionary relationship of the three variants with other IMPs. Then, a detailed sequence comparison of three groups of nine AGEs (including six *bla*_IMP_-carrying AGEs sequenced in this study) was performed. IMP genes are often situated within class 1 integrons harbored on broad-host-range plasmids or chromosomal AGEs. This work provides a deeper insight into the bioinformatics and epidemiology of IMP and *bla*_IMP_-carrying accessory genetic elements. The emergence of new IMP variants, and the diversity and complexity of their genetic environment make the prevention and control of drug-resistant strains more challenging, and clinical and public health management departments will need to pay attention to drug-resistant genes.

## MATERIALS AND METHODS

### Bacterial strains, identification, and detection of carbapenemase-encoding genes.

Five *bla*_IMP_-carrying clinical isolates (Table S1) were screened from nonduplicate carbapenem-resistant strains collected from different public hospitals in China. The presence of known MBL genes was investigated using PCR assays. Bacterial species identification was done using genome sequence-based average nucleotide identity analysis (http://www.ezbiocloud.net/tools/ani) (32). Bacterial antimicrobial susceptibility was tested by BioMérieux Vitek 2 and interpreted as per the Clinical and Laboratory Standards Institute (CLSI) guidelines (33).

### Sequencing and sequence assembly and annotation.

Bacterial genomic DNA was isolated using the UltraClean Microbial kit (Qiagen, Hilden, Germany) and sequenced from a paired-end library with an average insert size of 350 bp (range from 150 to 600 bp) on a HiSeq sequencer (Illumina, CA), as well as a shared DNA library with an average size of 15 kb (range from 10 to 20 kb) on a PacBio RSII sequencer (Pacific Biosciences, CA). The paired-end short Illumina reads were used to correct the long PacBio reads with the software proovread (34), and then corrected PacBio reads were assembled *de novo* using SMARTdenovo (https://github.com/ruanjue/smartdenovo). The sequencing data were checked using NanoPack29 (35) and FastQC (https://www.bioinformatics.babraham.ac.uk/projects/fastqc). Further sequence data mining was performed as described previously (36–38).

### Phylogenetic analysis.

Amino acid sequences were aligned using Clustal Omega 1.2.2 (39), and a neighbor-joining phylogenetic tree was constructed from aligned sequences using MEGA X 10.2 (40) with a bootstrap iteration of 1,000. The phylogenetic tree was subsequently visualized and modified using iTOL version 6 (https://itol.embl.de/).

### Cloning experiments and MIC measurements.

The *bla*_IMP-89/91/96_ coding regions together with their 450-bp upstream (promoter) regions and 300-bp downstream (terminator) regions from strains NY5709/NY3045/NY11291, respectively, were cloned into the cloning vector pUC57-Kan (pUC57K). Similarly, the coding region of each of the other *bla*_IMP_ variants together with the above promoter-proximal regions and terminator-proximal regions were synthesized and cloned into pUC57K. Each resulting recombinant plasmid was transformed through electroporation into E. coli TOP10, generating the relevant electroporant. Electroporant selection was done with 4 μg/mL meropenem (for *bla*_IMP_). Bacterial antimicrobial susceptibility was tested using the classic broth microdilution method, and interpreted as per the 2020 CLSI guidelines (33).

### Data availability.

The complete sequences of plasmids pNY5709-IMP and pNY11382-IMP, and those of the chromosomes of strains NY11291, NY3045, NY5710, and NY5709 were submitted to GenBank under accession numbers MN961670, CP097104, CP096975, CP059995, CP045554, and CP045551, respectively. The GenBank accession numbers of *bla*_IMP-89_, *bla*_IMP-91_, and *bla*_IMP-96_ are NG_070738, NG_076634, and NG_080776, respectively.
