# Supplementary material for: Characterization of Three Novel IMP Metallo-β-Lactamases, IMP-89, IMP-91, and IMP-96, and Diverse blaIMP-Carrying Accessory Genetic Elements from Chinese Clinical Isolates
Source: Microbiol Spectr. 2023 Apr 19;11(3):e04986-22. doi: 10.1128/spectrum.04986-22 (PMC10269577; doi:10.1128/spectrum.04986-22)
Supplement: Supplemental file 1 — Supplemental material. Download spectrum.04986-22-s0001.pdf, PDF file, 5.2 MB [file spectrum.04986-22-s0001.pdf]

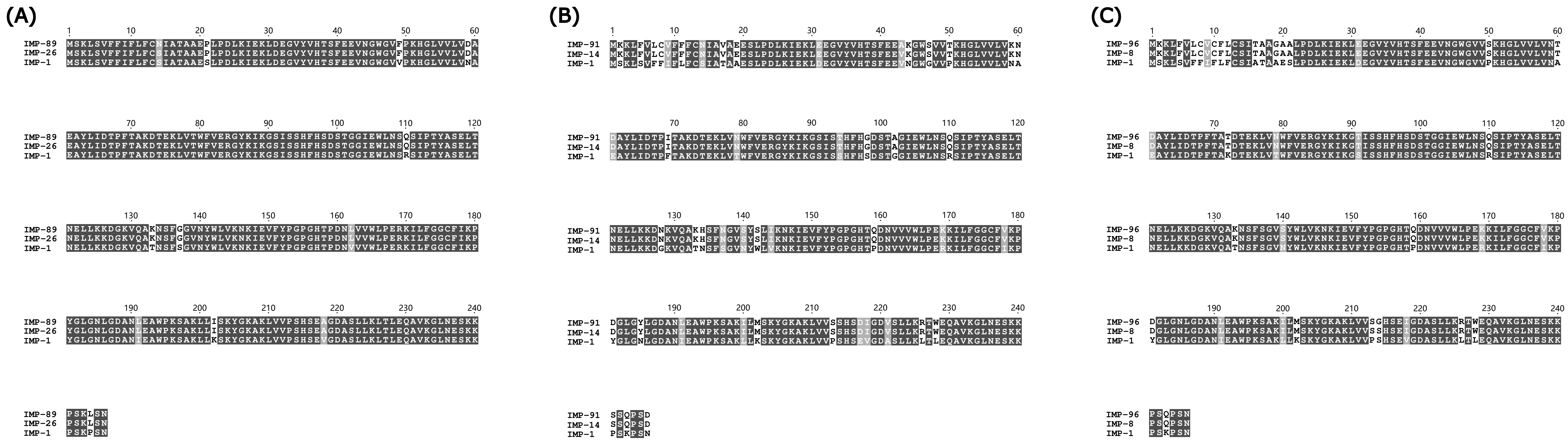

Fig. S1 Alignment of the amino acid sequences of some representative members of the IMP family.

(A) IMP-89 differed from IMP-26 by an amino acid substitution in the non-translated region (14 in IMP-1).

(B) IMP-91 differed from IMP-14 by an amino acid substitution Val61Ala (43 in IMP-1).

(C) IMP-96 differed from IMP-8 by an amino acid substitution Ser262Gly (214 in IMP-1).

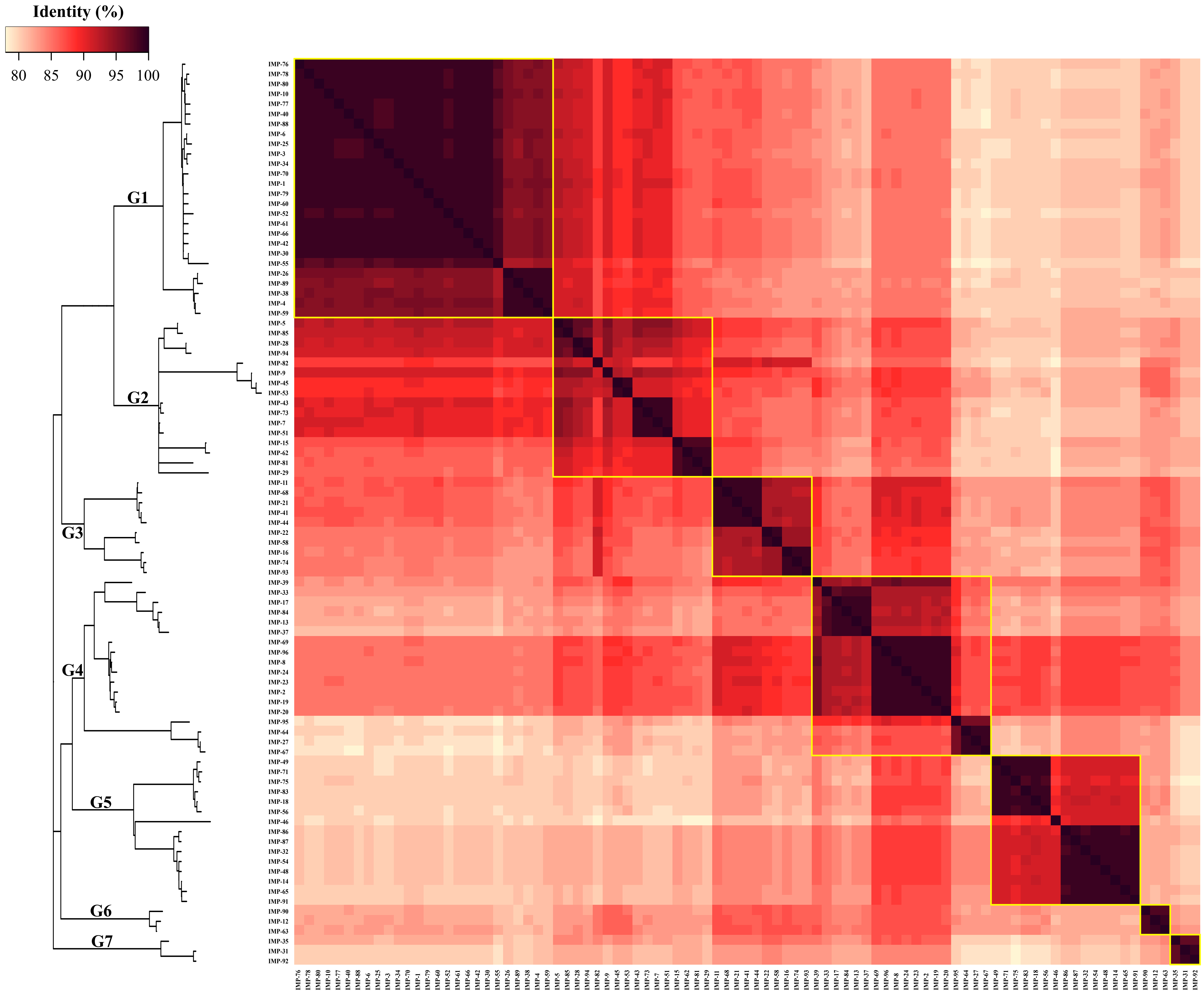

Fig. S2 A heatmap of pairwise comparison of the IMP type MBLs sequences.

Original data are shown in Table S3.

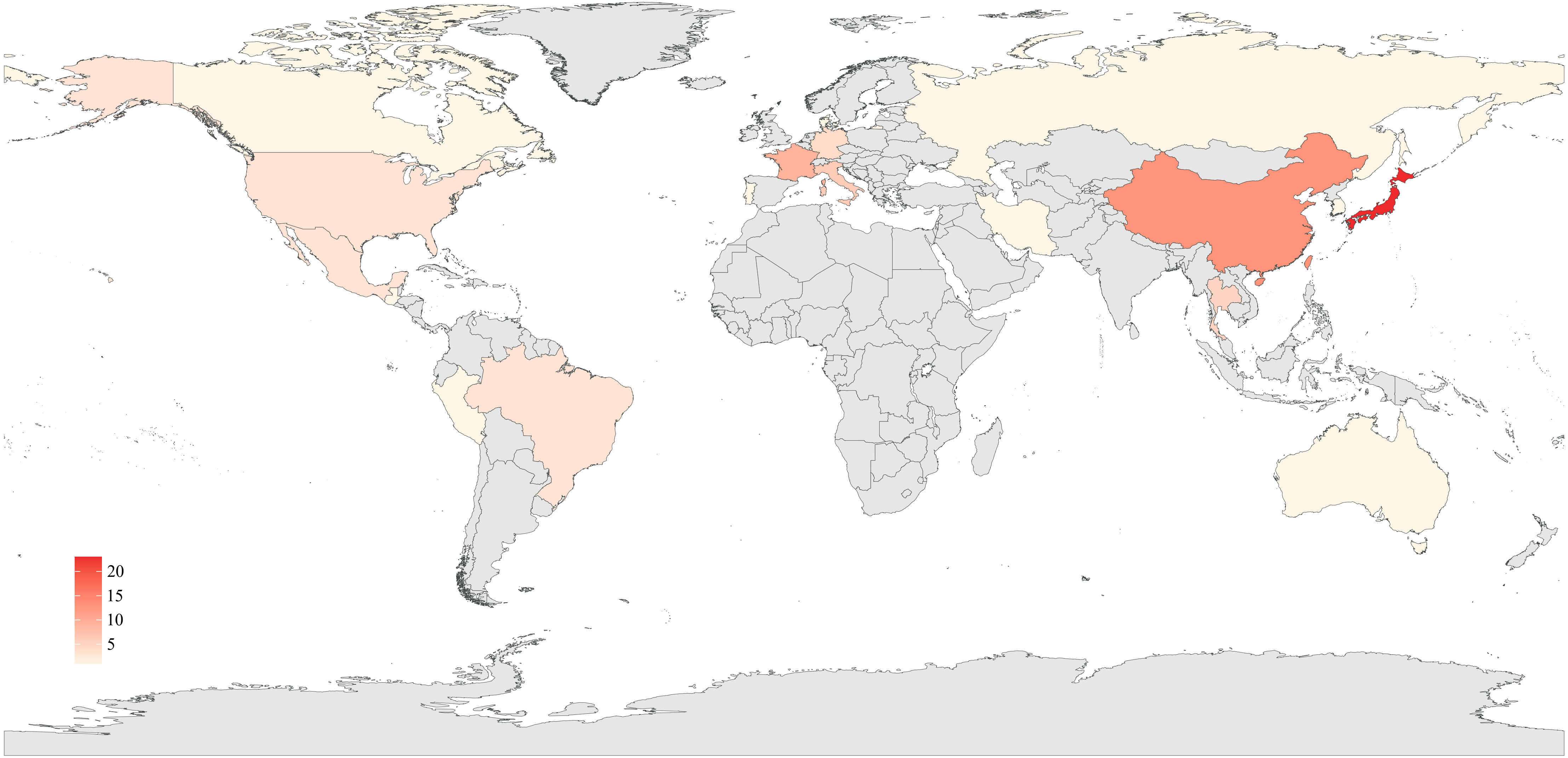

Fig. S3 Countries of the first report of IMP variants. Original data are shown in Table S2.

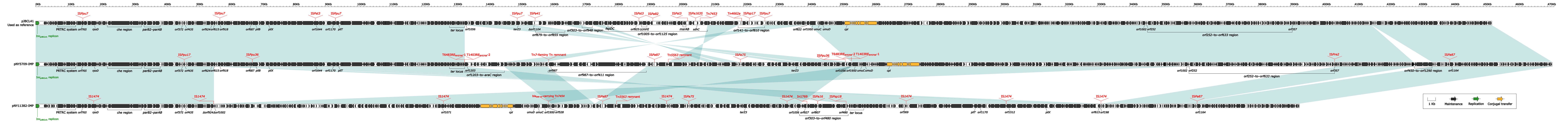

Fig. S4 Linear comparison of three Inc$_{pJBCL41}$ plasmids. Genes are denoted by arrows. Genes, AGEs, and other features are colored based on their functional classification. Shading denotes regions of homology (nucleotide identity ≥ 90%). Red characters represent accessory modules.

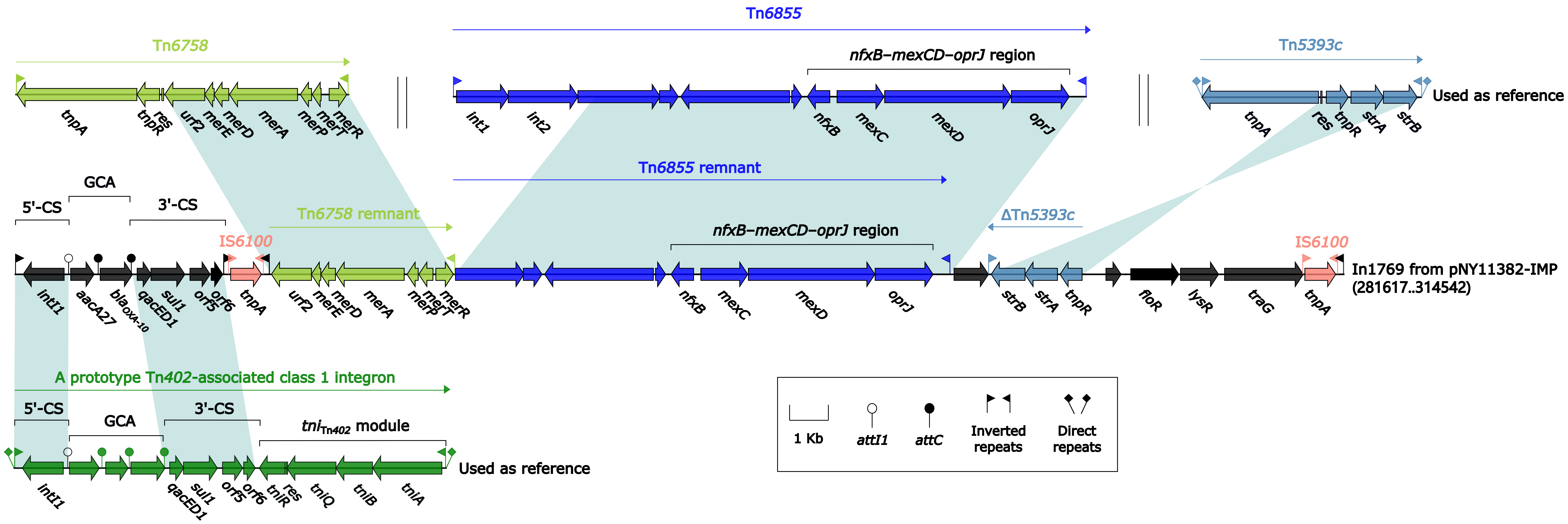

Fig. S5 Organization of In1769 from pNY11382-IMP.

Genes are denoted by arrows. Genes, AGEs, and other features are colored based on their functional classification. Shading denotes regions of homology (nucleotide identity ≥90%).

Numbers in brackets indicate nucleotide positions within the corresponding plasmid. The accession numbers of Tn*6758*, Tn*6855*, and Tn*5393c* used as references are JX448550, MK347425, and AF262622, respectively.

Table S1. Background information of the the five IMP-producing isolates charaterized in this study

Isolate	specie	Province	Hospital	Year	Specimen	Designation of chromosome and plasmids	Accession number
NY5709	*Pseudomonas putida*	Hunan	H02	2015	Urine	cNY5709	CP045551
						pNY5709-IMP	MN961670
						p420352-strA	MT074087
NY11382	*Pseudomonas sp.*	Beijing	H01	2011	Airway	cNY11382	CP097103
						pNY11382-IMP	CP097104
						pNY11382-NR	CP097105
						p24845-TEM	MF344572
NY11291	*Stenotrophomonas sp.*	Beijing	H01	2011	Urine	cNY11291	CP096975
NY3045	*Pseudomonas aeruginosa*	Jiangsu	H03	2015	Blood	cNY3045	CP059995
						pNY3045-NR	CP106923
NY5710	*Pseudomonas sp.*	Hunan	H02	2014	Airway	cNY5710	CP045554

Table S1. (continued)

Illumina Sequencing								
**Isolate**	**Sequencing**	**Q20 (%)**	**Q30 (%)**	**Depth (X)**	**Assembled bases (bp)**	**Numbers of contigs**	**Contig N50 (bp)**	**%GC content**
NY5709	Yes	97.53	93.23	400.44	6,805,944	135	173,924	61.02
NY11382	Yes	98.08	94.14	190.70	6,184,231	155	101,447	62.21
NY11291	Yes	97.99	94.10	256.40	4,929,145	34	445,323	66.07
NY3045	Yes	96.79	91.64	186.71	7,063,497	91	519,149	65.81
NY5710	Yes	97.92	94.22	283.99	5,545,712	60	229,324	62.35

Table S1. (continued)

PacBio Sequencing								
**Isolate**	**Designation of chromosome and plasmids**	**Sequencing**	**Number of Reads**	**Mean Read Length (bp)**	**Number of Bases (bp)**	**Reads N50 (bp)**	**Depth (X)**	**Assembled bases (bp)**
NY5709	cNY5709	Yes	071,851	13,211	949,225,729	17,087	136.46	6,165,619
	pNY5709-IMP							636,818
	p420352-strA							153,678
NY11382	cNY11382	Yes	394,929	7,978	3,150,972,577	9,224	499.31	5,681,002
	pNY11382-IMP							480,951
	pNY11382-NR							148,732
NY11291	cNY11291	Yes	220,595	8,712	1,921,981,309	10,092	387.36	4,961,805
NY3045	cNY3045	Yes	90,670	7,031	637,503,478	9,993	89.24	7,092,673
	pNY3045-NR							50,945
NY5710	cNY5710	Yes	71,000	13,761	977,048,370	18,005	174.07	5612,807

Table S1. (continued)

Minimum inhibitory concentration (µg/ml)/Antimicrobial susceptibility									
**Isolate**	**ticarcillin-clavulanate**	**piperacillin-tazobactam**	**ceftazidime**	**cefpoerazone-sulbactam**	**cefepime**	**aztreonam**	**imipenem**	**meropenem**	**amikacin**
NY5709	>= 128/R	>= 128/R	>= 64/R	>= 64/R	>= 32/R		>= 16/R	>= 16/R	<= 2/S
NY11382	>= 128/R	>= 128/R	>= 64/R	>= 64/R	>= 32/R		>= 16/R	>= 16/R	<= 2/S
NY11291									
NY3045	>= 128/R	>= 128/R	>= 64/R		>= 32/R		>= 16/R	>= 16/R	>= 64/R
NY5710	>= 128/R	8/S	>= 64/R	>= 64/R	>= 32/R		>= 16/R	>= 16/R	<= 2/S

Minimum inhibitory concentration (µg/ml)/Antimicrobial susceptibility								
**Isolate**	**tobramycin**	**ciprofloxacin**	**levofloxacin**	**doxycycline**	**minocycline**	**tigecycline**	**polymyxin**	**trimethoprim-sulfamethoxazole**
NY5709	>= 16/R	>= 4/R	>= 8/R	>= 16/R	4/S	4/I		
NY11382	8/I	>= 4/R	>= 8/R	4/S	8/I	>= 8/R		>= 320/R
NY11291			>= 8/R					>= 320/R
NY3045	>= 16/R	>= 4/R	>= 8/R			>= 8/R		
NY5710	<= 1/S	>= 4/R	>= 8/R	2/S	2/S	>= 8/R		80/R

Table S2. List of all the available 91 IMP alleles

Protein	Bacterial species	Strain name	Distribution	Accession number
IMP-1	*Serratia marcescens*		Japan	WP_003159548
IMP-2	*Acinetobacter baumannii*	AC-54/97	Italy	WP_063860578
IMP-3	*Shigella flexneri*	JS19622	Japan	WP_063860588
IMP-4	*Acinetobacter baumannii*		China	WP_015060105
IMP-5	*Acinetobacter baumannii*		Portugal	WP_063860611
IMP-6	*Serratia marcescens*		Japan	WP_032492096
IMP-7	*Pseudomonas aeruginosa*		Canada	WP_063860619
IMP-8	*Klebsiella pneumoniae*	KP-0787	China	WP_012695457
IMP-9	*Pseudomonas aeruginosa*	PA96	China	WP_021018609
IMP-10	*Pseudomonas aeruginosa*	PAI134	Japan	WP_032490175
IMP-11	*Acinetobacter baumannii*	ABI1	Japan	WP_063860573
IMP-12	*Pseudomonas putida*	758/00	Italy	WP_063860574
IMP-13	*Pseudomonas aeruginosa*	86-14571A	Italy	WP_042862936
IMP-14	*Pseudomonas aeruginosa*		Thailand	WP_039819893
IMP-15	*Pseudomonas aeruginosa*		Thailand	WP_063860575
IMP-16	*Pseudomonas aeruginosa*	101-4704C	Brazil	WP_063860576
IMP-17	*Pseudomonas aeruginosa*	Pa-7/02	Italy	WP_063860577
IMP-18	*Pseudomonas aeruginosa*		United States	WP_063848609
IMP-19	*Aeromonas caviae*	A324R	France	WP_032491874
IMP-20	*Pseudomonas aeruginosa*	MRY04-1316	Japan	WP_063860579
IMP-21	*Pseudomonas aeruginosa*	NCB03-104	Japan	WP_063860580
IMP-22	*Pseudomonas fluorescens*		Italy	WP_063860581
IMP-23	*Citrobacter freundii*	HSHCF-50	China	WP_063860582

IMP-24	*Serratia marcescens*	sm177-6	China	WP_063860583
IMP-25	*Pseudomonas aeruginosa*	KMP0701	Korea	WP_063860584
IMP-26	*Pseudomonas aeruginosa*	DR26420/08	Singapore	WP_060614779
IMP-27	*Proteus mirabilis*	PmCB1	United States	WP_063860585
IMP-28	*Klebsiella oxytoca*	11005	Spanish	WP_063860586
IMP-29	*Pseudomonas aeruginosa*	BES10298	France	WP_063860587
IMP-30	*Pseudomonas aeruginosa*	3107	Russia	WP_063860589
IMP-31	*Pseudomonas aeruginosa*	NRZ-00156	Germany	WP_031943232
IMP-32	*Klebsiella pneumoniae*	KP-PNK-1	Thailand	WP_063860590
IMP-33	*Pseudomonas aeruginosa*	85-7090A	Italy	WP_063860591
IMP-34	*Klebsiella oxytoca*	MS5279	Japan	WP_032492622
IMP-35	*Pseudomonas aeruginosa*	1876	Germany	WP_063860592
IMP-37	*Pseudomonas aeruginosa*	CNR 06-BRE	French	WP_063860593
IMP-38	*Klebsiella pneumoniae*	KP9	China	WP_063860594
IMP-39	*Pseudomonas aeruginosa*	17-5136	France	WP_136512068
IMP-40	*Pseudomonas aeruginosa*	JICC 55824	Japan	WP_063860598
IMP-41	*Pseudomonas aeruginosa*	JICC 55826	Japan	WP_063860601
IMP-42	*Acinetobacter soli*	JICC 55015	Japan	WP_063860603
IMP-43	*Pseudomonas aeruginosa*	NCGM 1496	Japan	WP_063860606
IMP-44	*Pseudomonas aeruginosa*	NCGM 1663	Japan	WP_063860607
IMP-45	*Pseudomonas aeruginosa*	M140A	China	WP_063860608
IMP-46	*Pseudomonas putida*	13-1694	France	WP_136512069
IMP-48	*Pseudomonas aeruginosa*	926467		WP_063860609
IMP-49	*Pseudomonas aeruginosa*	1128657	Brazil	WP_063860610
IMP-51	*Pseudomonas aeruginosa*	NCGM 3025	Viet Nam	WP_063860612

IMP-52	*Escherichia coli*	TUM14831	Japan	WP_063860613
IMP-53	*Pseudomonas aeruginosa*	NF812166		WP_063860614
IMP-54	*Pseudomonas aeruginosa*	750109	Thailand	WP_063860615
IMP-55	*Acinetobacter baumannii*	56	Iran	WP_063860616
IMP-56	*Pseudomonas aeruginosa*	1207312	Guatemala	WP_063860617
IMP-58	*Pseudomonas putida*	CPO20150081	Denmark	WP_063860618
IMP-59	*Escherichia coli*	1216811	Australia	WP_094009805
IMP-60	*Enterobacter cloacae*	NCGM-ECl242	Japan	WP_065102288
IMP-61	*Acinetobacter baumannii*	NRZ-26903		WP_065419570
IMP-62	*Pseudomonas aeruginosa*	PE83	Mexico	WP_069280710
IMP-63	*Pseudomonas aeruginosa*	1156	France	WP_071593225
IMP-64	*Proteus mirabilis*	20299C	United States	WP_071593226
IMP-65	*Pseudomonas aeruginosa*	MPPA-138	Thailand	WP_150823469
IMP-66	*Escherichia coli*	NR336	Japan	WP_085562391
IMP-67	*Providencia rettgeri*	Put_Imp_1		WP_088245214
IMP-68	*Klebsiella pneumoniae*	TA6363	Japan	WP_096807443
IMP-69	*Providencia sp.*	WCHPr-ZXX99369	China	WP_099156051
IMP-70	*Pseudomonas aeruginosa*	NRZ-38503	Germany	WP_102607459
IMP-71	*Pseudomonas aeruginosa*	174324	France	WP_104009852
IMP-73	*Pseudomonas aeruginosa*	1739129	Japan	WP_109545053
IMP-74	*Pseudomonas aeruginosa*	1714568	Brazil	WP_109791207
IMP-75	*Pseudomonas aeruginosa*	1641818	Mexico	WP_109791208
IMP-76	*Pseudomonas aeruginosa*	NCGM 3689	Japan	WP_114699280
IMP-77	*Pseudomonas aeruginosa*	NCGM 3799	Japan	WP_114699281
IMP-78	*Pseudomonas aeruginosa*	NCGM 3848	Japan	WP_114699282

IMP-79	*Pseudomonas aeruginosa*	163874	France	WP_116786839
IMP-80	*Pseudomonas aeruginosa*	NCGM 3336	Japan	WP_122630861
IMP-81	*uncultured bacterium*		Indian	WP_148044415
IMP-82	*Pseudomonas aeruginosa*	NRZ-43907	Germany	WP_148044416
IMP-83	*Pseudomonas aeruginosa*	1767579	Mexico	WP_148044417
IMP-84	*Pseudomonas aeruginosa*	1849458		WP_148044418
IMP-85	*Pseudomonas aeruginosa*	197018	France	WP_152315465
IMP-86	*Pseudomonas aeruginosa*	KM2018-19	China	WP_219860708
IMP-87	*Pseudomonas aeruginosa*	KM2018-38	China	WP_219860709
IMP-88	*Pseudomonas aeruginosa*	JUPA4263	Japan	WP_188331870
IMP-89	*Pseudomonas putida*	NY5709	China	WP_181727105
IMP-90	*Pseudomonas aeruginosa*	NRZ-62848		WP_213994587
IMP-91	*Pseudomonas aeruginosa*	NY3045	China	WP_079387329
IMP-92	*Pseudomonas aeruginosa*	A212111		WP_240067720
IMP-93	*Pseudomonas aeruginosa*	17404	Peru	WP_234855925
IMP-94	*Achromobacter xylosoxidans*	218832	France	WP_240067721
IMP-95	*Acinetobacter baumannii*	1J		WP_242934067
IMP-96	*Stenotrophomonas sp.*	NY11291	China	WP_186931965

>IMP-1

MSKLSVFFIFLFCSIATAAESLPDLKIEKLDEGVYVHTSFEEVNGWGVVPKHGLVVLVNAEAYLIDTPFTAKDTEKLVTWFVERGYKIKGSISSHFHSDSTGGIEWLNS
RSIPTYASELTNELLKKDGKVQATNSFSGVNYWLVKNKIEVFYPGPGHTPDNVVVWLPERKILFGGCFIKPYGLGNLGDANIEAWPKSAKLLKSKYGKAKLVVPSHS
EVGDASLLKLTLEQAVKGLNESKKPSKPSN

>IMP-2

MKKLFVLCVCFLCSITAAGARLPDLKIEKLEEGVYVHTSFEEVNGWGVVSKHGLVVLVNTDAYLIDTPFTATDTEKLVNWFVERGYKIKGTISSHFHSDSTGGIEWL
NSQSIPTYASELTNELLKKDGKVQAKNSFSGVSYWLVKNKIEVFYPGPGHTQDNVVVWLPEKKILFGGCFVKPDGLGNLGDANLEAWPKSAKILMSKYVKAKLVV
SSHSEIGDASLLKRTWEQAVKGLNESKKPSQPSN

>IMP-3
MSKLSVFFIFLFCSIATAAESLPDLKIEKLDEGVYVHTSFEEVNGWGVVPKHGLVVLVNAEAYLIDTPFTAKDTEKLVTWFVERGYKIKGSISSHFHSDSTGGIGWLNS
RSIPTYASELTNELLKKDGKVQATNSFSGVNYWLVKNKIEVFYPGPGHTPDNVVVWLPERKILFGGCFIKPYGLGNLGDANIEAWPKSAKLLKSKYGKAKLVVPGH
SEVGDASLLKLTLEQAVKGLNESKKPSKPSN

>IMP-4
MSKLSVFFIFLFCSIATAAEPLPDLKIEKLDEGVYVHTSFEEVNGWGVVPKHGLVVLVDAEAYLIDTPFTAKDTEKLVTWFVERGYKIKGSISSHFHSDSTGGIEWLNS
QSIPTYASELTNELLKKDGKVQAKNSFGGVNYWLVKNKIEVFYPGPGHTPDNLVVWLPERKILFGGCFIKPYGLGNLGDANLEAWPKSAKLLISKYGKAKLVVPSH
SEAGDASLLKLTLEQAVKGLNESKKPSKLSN

>IMP-5
MSKLFVFFMFLFCSITAAAESLPDLKIEKLDEGVYVHTSFEEVNGWGVVPKHGLVVLVNTEAYLIDTPFTAKDTEKLVTWFVERGYKIKGSISSHFHSDSTGGIEWLN
SQSIPTYASELTNELLKKDGKVQAKNSFSGASYWLVKKKIEVFYPGPGHTPDNVVVWLPENRVLFGGCFVKPYGLGNLGDANVEAWPKSAKLLMSKYGKAKLVVP
SHSEVGDASLLKRTLEQAVKGLNESKKPSKPSN

>IMP-6
MSKLSVFFIFLFCSIATAAESLPDLKIEKLDEGVYVHTSFEEVNGWGVVPKHGLVVLVNAEAYLIDTPFTAKDTEKLVTWFVERGYKIKGSISSHFHSDSTGGIEWLNS
RSIPTYASELTNELLKKDGKVQATNSFSGVNYWLVKNKIEVFYPGPGHTPDNVVVWLPERKILFGGCFIKPYGLGNLGDANIEAWPKSAKLLKSKYGKAKLVVPGH
SEVGDASLLKLTLEQAVKGLNESKKPSKPSN

>IMP-7
MKKLSVFFMFLFCSIAASGEALPDLKIEKLDEGVYVHTSFEEVNGWGVVPKHGLVVLVNTDAYLIDTPFTAKDTEKLVTWFVERGYKIKGSISSHFHSDSTGGIEWL

NSQSIPTYASELTNELLKKDGKVQAKNSFSGASYWLVKKKIEIFYPGPGHTPDNVVVWLPEHRVLFGGCFVKPYGLGNLGDANLEAWPKSAKLLVSKYGKAKLVVP
SHSEVGDASLLKRTLEQAVKGLNESKKLSKPSN

>IMP-8
MKKLFVLCVCFLCSITAAGAALPDLKIEKLEEGVYVHTSFEEVNGWGVVSKHGLVVLVNTDAYLIDTPFTATDTEKLVNWFVERGYKIKGTISSHFHSDSTGGIEWL
NSQSIPTYASELTNELLKKDGKVQAKNSFSGVSYWLVKNKIEVFYPGPGHTQDNVVVWLPEKKILFGGCFVKPDGLGNLGDANLEAWPKSAKILMSKYGKAKLVV
SSHSEIGDASLLKRTWEQAVKGLNESKKPSQPSN

>IMP-9
MSKLFVFFMFLFCSITAAGESLPDLKIEKLDEGVYVHTSFEEVNGWGVIPKHGLVVLVNTDAYLIDTPFTAKDTENLVNWFVERGYRIKGSISSHFHSDSTGGIEWLN
SQSIPTYASELTNELLKKDGKVQAKYSFSGVSYWLVKKKIEVFYPGPGHAPDNVVVWLPENRVLFGGCFVKPYGLGNLGDANLEAWPKSAKLLMSKYSKAKLVVP
SHSDIGDSSLLKLTWEQTVKGFNESKKSTTAH

>IMP-10
MSKLSVFFIFLFCSIATAAESLPDLKIEKLDEGVYVHTSFEEVNGWGVFPKHGLVVLVNAEAYLIDTPFTAKDTEKLVTWFVERGYKIKGSISSHFHSDSTGGIEWLNS
RSIPTYASELTNELLKKDGKVQATNSFSGVNYWLVKNKIEVFYPGPGHTPDNVVVWLPERKILFGGCFIKPYGLGNLGDANIEAWPKSAKLLKSKYGKAKLVVPSHS
EVGDASLLKLTLEQAVKGLNESKKPSKPSN

>IMP-11
MKKLFVLCIFLFCSITAAGASLPDLKIEKLEEGVYVHTSFEEVNGWGVVSKHGLVVLVNTDAYLIDTPFTAKDTEKLVNWFVERGYKIKGSISSHFHSDSTGGIEWLN
SQSIPTYASVLTNELLKKDGKVQAKNSFSGVSYWLVKNKIEVFYPGPGHTQDNVVVWLPKNKILFGGCFVKPYGLGNLDDANVEAWPHSAEKLISKYGNAKLVVP
SHSDIGDASLLKLTWEQAVKGLNESKKSNTVH

>IMP-12
MKKLFVLCIFLFLSITASGEVLPDLKIEKLEEGVYLHTSFEEVSGWGVVTKHGLVVLVNNDAYLIDTPFTNKDTEKLVAWFVGRGFTIKGSVSSHFHSDSTGGIEWLN
SQSIPTYASELTNELLKKNGKVQATNSFSGVSYWLVKNKIEIFYPGPGHTQDNVVVWLPENKILFGGCFVKPDGLGNLDDANLKAWPKSAKILMSKYGKAKLVVSG

HSEIGNASLLKLTWEQAVKGLKESKKPLLPSN

>IMP-13
MKKLFVLCVCFFCSITAAGAALPDLKIEKLEEGVFVHTSFEEVNGWGVVTKHGLVVLVNTDAYLIDTPFTATDTEKLVNWFVERGYEIKGTISSHFHSDSTGGIEWLN
SQSIPTYASELTNELLKKSGKVQAKYSFSEVSYWLVKNKIEVFYPGPGHTQDNLVVWLPESKILFGGCFIKPHGLGNLGDANLEAWPKSAKILMSKYGKAKLVVSSH
SEKGDASLMKRTWEQALKGLKESKKTSSPSN

>IMP-14
MKKLFVLCVFFFCNIAVAEESLPDLKIEKLEEGVYVHTSFEEVKGWSVVTKHGLVVLVKNDAYLIDTPITAKDTEKLVNWFVERGYKIKGSISTHFHGDSTAGIEWLN
SQSIPTYASELTNELLKKDNKVQAKHSFNGVSYSLIKNKIEVFYPGPGHTQDNVVVWLPEKKILFGGCFVKPDGLGYLGDANLEAWPKSAKILMSKYGKAKLVVSS
HSDIGDVSLLKRTWEQAVKGLNESKKSSQPSD

>IMP-15
MNKLSVFFMFMFCSITAAGESLPDLKIEKLDEGVYVHTSFEEVNGWGVVPKHGLVVLVNTEAYLIDTPFTAKDTEKLVTWFVERGYKIKGSISSHFHSDSTGGIEWL
NSQSIPTYASELTNELLKKDGKVQAKNSFSGGSYWLVNNKIEVFYPGPGHTPDNVVVWLPENRVLFGGCFVKPYGLGNLGDANLEAWPKSAKILMSKYGKAKLVV
SSHSETGNASLLKLTWEQAVKGLKESKKPSLPSN

>IMP-16
MKKLFVLCIFLFCSITAAGESLPDLKIEKLEDGVYVHTSFEEVNGWGVVTKHGLVFLVNTDAYLIDTPFAAKDTEKLVNWFVERGYKIKGSISSHFHSDSSGGIEWLN
SQSIPTYASELTNELLKKNGKVQAKNSFSGVSYWLLKNKIEIFYPGPGHTQDNVVVWLPEKKILFGGCFVKPYGLGNLDDANVEAWPHSAEILMSRYGNAKLVVPS
HSDVGDASLLKLTWEQAVKGLKESKKPSQPSN

>IMP-17
MKKLFVLCVCFFCSITAAGAALPDLKIEKLEEGVFVHTSFEEVNGWGVVTKHGLVVLVNTDAYLIDTPFTATDTEKLVNWFVERGYEIKGTISSHFHSDSTGGIEWLN
SQSIPTYASELTNELLKKSGKVQAKYSFSGVSYWLVKNKIEVFYPGPGHTQDNLVVWLPESEILFGGCFIKPHGLGNLGDANLEAWPKSAKILMSKYGKAKLVVSSH
SEKGDASLMKRTWEQALKGLKESKKTSSPSN

>IMP-18
MKKLFVLCVFFLCNIAAADDSLPDLKIEKLEKGVYVHTSFEEVKGWGVVTKHGLVVLVKNDAYLIDTPITAKDTEKLVNWFIEHGYRIKGSISTHFHGDSTAGIEWL
NSQSISTYASELTNELLKKDNKVQATNSFSGVSYSLIKNKIEVFYPGPGHTQDNVVVWLPEKKILFGGCFVKPDGLGNLGDANLEAWPKSAKILMSKYGKAKLVVSS
HSEIGNASLLQRTWEQAVKGLNESKKPLQPSS

>IMP-19
MKKLFVLCVCFLCSITAAGAALPDLKIEKLEEGVYVHTSFEEVNGWGVVSKHGLVVLVNTDAYLIDTPFTATDTEKLVNWFVERGYKIKGTISSHFHSDSTGGIEWL
NSQSIPTYASELTNELLKKDGKVQAKNSFSGVSYWLVKNKIEVFYPGPGHTQDNVVVWLPEKKILFGGCFVKPDGLGNLGDANLEAWPKSAKILMSKYVKAKLVV
SSHSEIGDASLLKRTWEQAVKGLNESKKPSQPSN

>IMP-20
MKKLFVLCVCFLCSITAAGAALPDLKIEKLEEGVYVHTSFEEVNGWGVFSKHGLVVLVNTDAYLIDTPFTATDTEKLVNWFVERGYKIKGTISSHFHSDSTGGIEWL
NSQSIPTYASELTNELLKKDGKVQAKNSFSGVSYWLVKNKIEVFYPGPGHTQDNVVVWLPEKKILFGGCFVKPDGLGNLGDANLEAWPKSAKILMSKYVKAKLVV
SSHSEIGDASLLKRTWEQAVKGLNESKKPSQPSN

>IMP-21
MKKLFVLCIFLFCSITAAGASLPDLKIEKLEEGVYVHTSFEEVNGWGVASKHGLVVLVNTDAYLIDTPFTAKDTEKLVNWFVERGYKIKGSISSHFHSDSTGGIEWLN
SQSIPTYASVLTNELLKKDGKVQAKNSFSGVSYWLVKNKIEVFYPGPGHTQDNVVVWLPKNKILFGGCFVKPYGLGNLDDANVEAWPHSAEKLISKYGNAKLVVP
SHSDIGDASLLKLTWEQAVKGLNESKKSNTVH

>IMP-22
MKKLFVLCVFLFCSITAAGESLPDLKIEKLEEGVYVHTSFEEVNGWGVVSKHGLVILVNTDAYLIDTPFTAKDTEKLVTWFVERGYKIKGSISSHFHSDSTGGIEWLN
SQSIPTYASELTNDLLKQNGKVQAKNSFSGVSYWLVKNKIEVFYPGPGHTQDNVVVWLPEKKILFGGCFVKPYGLGNLDDANVVAWPHSAEILMSRYGNAKLVVP
SHSDIGDASLLKLTWEQAVKGLKESKKPSEPSN

>IMP-23
MKKLFVLCVCFLCSITAAGAALPDLKIEKLEEGVYVHTSFEEVNGWGVFSKHGLVVLVNTDAYLIDTPFTATDTEKLVNWFVERGYKIKGTISSHFHSDSTGGIEWL
NSQSIPTYASELTNELLKKDGKVQAKNSFSGVSYWLVKNKIEVFYPGPGHTQDNVVVWLPEKKILFGGCFVKPDGLGNLGDANLEAWPKSAKILMSKYGKAKLVV
SSHSEIGDASLLKRTWEQAVKGLNESKKPSQPSN

>IMP-24
MKKLFVLCVCFLCSITAAGAALPDLKIEKLEEGVYVHTSFEEVNGWGVVSKHGLVVLVNTDAYLIDTPFTATDTEKLVNWFVERGYKIKGTISSHFHSDSTGGIEWL
NSQSIPTYASELTNELLKKDGKVQAKNSFSGVSYWLVKNKIEVFYPGPGHTQDNVVVWLPEKKILFGGCFVKPDGLGNLGDANLEAWPKSAKILMSKYGKAKLVV
SSHSEIGDASLLKRTWEQAVKGLNESRKPSQPSN

>IMP-25
MSKLSVFFIFLFCSIATAAESLPDLKIEKLDEGVYVHTSFEEVNGWGVVPKHGLVVLVNAEAYLIDTPFTAKDTEKLVTWFVERGYKIKGSISSHFHSDSTGGIEWLNS
RSIPTYASELTNELLKKDGKVQATNSFSGVNYWLVKNKIEVFYPGPGHTPDNVVVWLPERKILFGGCFIKPYGLGNLSDANIEAWPKSAKLLKSKYGKAKLVVPGHS
EVGDASLLKLTLEQAVKGLNESKKPSKPSN

>IMP-26
MSKLSVFFIFLFCSIATAAEPLPDLKIEKLDEGVYVHTSFEEVNGWGVFPKHGLVVLVDAEAYLIDTPFTAKDTEKLVTWFVERGYKIKGSISSHFHSDSTGGIEWLNS
QSIPTYASELTNELLKKDGKVQAKNSFGGVNYWLVKNKIEVFYPGPGHTPDNLVVWLPERKILFGGCFIKPYGLGNLGDANLEAWPKSAKLLISKYGKAKLVVPSH
SEAGDASLLKLTLEQAVKGLNESKKPSKLSN

>IMP-27
MKKLFVLCVFVFCSITVAGETLPNLRVEKLEEGVYVHTSYEEVKGWGVVTKHGLVVLIGADAYLIDTPFTAKDTEKLVNWFVERGYKIKGTVSSHFHSDSTGGIEW
LNSQSIPTYASELTNELLKKDGKVQAKNSFDGVSYWLAKDKIEVFYPGPGHTQDNVVVWLPEKEILFGGCFVKPHGLGNLGDANLEAWPESAKILMEKYGKAKLV
VSGHSETGDATHLKRTWEQAVKGLKESKKTLQPSN

>IMP-28

MSKLFVFFMFLFCSITAAAESLPDLKIERLDEGVYVHTSFEEVNGWGVVPKHGLVVLVNTEAYLIDTPFTAKDTEKLVTWFVGRGYKIKGSISSHFHSDSTGGIEWLN
SQSIPTYASELTNELLKKDGKVQAKNSFGGVSYWLVKNKIEVFYPGPGHTPDNVVVWLPENRVLFGGCFVKPYGLGNLGDANLEAWPKSAKLLMSKYGKAKLVV
PSHSEVGDASLLKRTLEHAVKGLNESKKPSKPSN

>IMP-29
MSKLFVFLIFLFCSITAAAESLPDLKIEKLDEGVYVHTSFEEVNGWGVVPKHGLVVLVNTEAYLIDTPFTAKDTEKLVTWFVERGYKIKGSISSHFHSDSTGGIEWLNS
QSIPTYASELTNELLKKGGKVQAKNSFSGVSYWLVKKKIEVFYPGPGHTPDNVVVWLPENRVLFGGCFVKPYGLGNLDDANVEAWPHSAEILMSRYGNAKLVVPS
HSDIGNASLLKLTWEQAVKGLKESKKPSQPSN

>IMP-30
MSKLSVFFIFLFCSIATAAESLPDLKIEKLDEGVYVHTSFKEVNGWGVVPKHGLVVLVNAEAYLIDTPFTAKDTEKLVTWFVERGYKIKGSISSHFHSDSTGGIEWLNS
RSIPTYASELTNELLKKDGKVQATNSFSGVNYWLVKNKIEVFYPGPGHTPDNVVVWLPERKILFGGCFIKPYGLGNLGDANIEAWPKSAKLLKSKYGKAKLVVPSHS
EVGDASLLKLTLEQAVKGLNESKKPSKPSN

>IMP-31
MKKIFVLFVFLFCSITAAGESLPDIKIEKLDEDVYVHTSFEKITGWGVITKHGLVVLVNTDAYIIDTPFTAKDTEKLVRWFVGRGYKIKGSISSHFHSDSAGGIEWLNSQ
SIPTYASKLTNELLKKNGNAQAENSFSGVSYWLVKHKIEVFYPGPGHTQDNVVVWLPEKKILFGGCFIKPDGLGYLGDANLEAWPKSAETLMSKYGNAKLVVSSHS
EIGGASLLKRTWEQAVKGLKESKNHHSPK

>IMP-32
MKKLFVLCVFFFCNIAVAEESLPDLKIEKLEEGVYVHTSFEEVKGWSVVTKHGLVVLVKNDAYLIDTPITAKDTEKLVNWFVERGYKIKGSISTHFHGDSTAGIEWLN
SQSIPTYASELTNELLKKDNKVQAKHSFYGVSYSLIKNKIEVFYPGPGHTQDNVVVWLPEKKILFGGCFVKPDGLGYLGDANLEAWPKSAKILMSKYGKAKLVVSS
HSDIGDVSLLKRTWEQAVKGLNESKKSSQPSD

>IMP-33
MKKLFVLCVCFFCSITAAGSSLPDLKIEKLEEGVFVHTSFEEVNGWGVVTKHGLVVLVNTDAYLIDTPFTATDTEKLVNWFVERGYKIKGTISSHFHSDSTGGIEWLN

SQSIPTYASELTNELLKKSGKVQAKYSFSEVSYWLVKNKIEVFYPGPGHTQDNLVVWLPESKILFGGCFVKPHGLGNLGDANLEAWPKSAKILMSKYGKAKLVVSS
HSEKGDASLLKRTWEQALKGLKESKKTSSPSN

>IMP-34
MSKLSVFFIFLFCSIATAAESLPDLKIEKLDEGVYVHTSFEEVNGWGVVPKHGLVVLVNAEAYLIDTPFTAKDTEKLVTWFVERGYKIKGSISSHFHSDSTGGIGWLNS
RSIPTYASELTNELLKKDGKVQATNSFSGVNYWLVKNKIEVFYPGPGHTPDNVVVWLPERKILFGGCFIKPYGLGNLGDANIEAWPKSAKLLKSKYGKAKLVVPSHS
EVGDASLLKLTLEQAVKGLNESKKPSKPSN

>IMP-35
MKKIFVLFVFLFCSITAAGESLPDIKIEKLDEDVYVHTSFEEDNGWGVITKHGLVVLVNTDAYIIDTPFTAKDTEKLVRWFVGRGYKIKGSISSHFHSDSAGGIEWLNS
QSIPTYASKLTNELLKKNGNAQAENSFSGVSYWLVKHKIEVFYPGPGHTQDNVVVWLPEKKILFGGCFIKPDGLGYLGDANLEAWPKSAETLMSKYGNAKLVVSS
HSEIGGASLLKRTWEQAVKGLKESKKPSQPNN

>IMP-37
MKKLFVLCVCFFCSITAAGAALPDLKIEKLEEGVFVHTSFEEVNGWGVVTKHGLVVLVNTDAYLIDTPFTATDTEKLVNWFVERGYEIKGTISSHFHSDSTGGIEWLN
SQSIPTYASELTNELLKKSGKVQAKYSFSEVSYWLVKNKIEVFYPGPGHTQDNLVVWLPESKILFGGCFIKPHGLGNLGDANLEAWPKSAKILMSKYGKAKLVVSSH
SEKGDASLMKRTWEQALKGLKESKKTSSQSTAS

>IMP-38
MSKLSVFFIFLFCSIATAAEPLPDLKIEKLDEGVYVHTSFEEVNGWGVVPKHGLVVLVDAEAYLIDTPFTAKDTEKLVTWFVERGYKIKGSISSHFHSDSTGGIEWLNS
QSIPTYASELTNELLKKDGKVQAKNSFGGVNYWLVKNKIEVFYPGPGHTPDNLVVWLPERKILFGGCFIKPYGLGNLGDANLEAWPKSAKLLISKYGKAKLVVPGH
SEAGDASLLKLTLEQAVKGLNESKKPSKLSN

>IMP-39
MKKLFVLCVCFLCSITAAGAALPDLKIEKLEEGVYVHTSFEEVNGWGVVSKHGLVVLVNTDAYLIDTPFTATDTEKLVNWFVERGYKIKGTISSHFHSDSTGGIEWL
NSQSIPTYASELTNELLKKDGKVQAKHSFSGVSYWLVKNKIEVFYPGPGHTQDNVVVWLPENKILFGGCFVKPHGLGNLGDANLEAWPKSAKILMSKYGKAKLVV

SSHSETGGASLLKRTWEQALKGLKESKKPSPPSN

>IMP-40
MSKLSVFFIFLFCSIATAAESLPDLKIEKLDEGVYVHTSFEEVNGWGVFPKHGLVVLVNAEAYLIDTPSTAKDTEKLVTWFVERGYKIKGSISSHFHSDSTGGIEWLNS
RSIPTYASELTNELLKKDGKVQATNSFSGVNYWLVKNKIEVFYPGPGHTPDNVVVWLPERKILFGGCFIKPYGLGNLGDANIEAWPKSAKLLKSKYGKAKLVVPSHS
EVGDASLLKLTLEQAVKGLNESKKPSKPSN

>IMP-41
MKKLFVLCIFLFCSITAAGASLPDLKIEKLEEGVYVHTSFEEVNGWGVFSKHGLVVLVNTDAYLIDTPFTAKDTEKLVNWFVERGYKIKGSISSHFHSDSTGGIEWLN
SQSIPTYASVLTNELLKKDGKVQAKNSFSGVSYWLVKNKIEVFYPGPGHTQDNVVVWLPKNKILFGGCFVKPYGLGNLDDANVEAWPHSAEKLISKYGNAKLVVP
SHSDIGDASLLKLTWEQAVKGLNESKKSNTVH

>IMP-42
MSKLSVFFIFLFCSIATAAESLPDLKIEKLDEGVYVHTSFEEVNRWGVVPKHGLVVLVNAEAYLIDTPFTAKDTEKLVTWFVERGYKIKGSISSHFHSDSTGGIEWLNS
RSIPTYASELTNELLKKDGKVQATNSFSGVNYWLVKNKIEVFYPGPGHTPDNVVVWLPERKILFGGCFIKPYGLGNLGDANIEAWPKSAKLLKSKYGKAKLVVPSHS
EVGDASLLKLTLEQAVKGLNESKKPSKPSN

>IMP-43
MKKLSVFFMFLFCSIAASGEALPDLKIEKLDEGVYVHTSFEEVNGWGVFPKHGLVVLVNTDAYLIDTPFTAKDTEKLVTWFVERGYKIKGSISSHFHSDSTGGIEWL
NSQSIPTYASELTNELLKKDGKVQAKNSFSGASYWLVKKKIEIFYPGPGHTPDNVVVWLPEHRVLFGGCFVKPYGLGNLGDANLEAWPKSAKLLVSKYGKAKLVVP
SHSEVGDASLLKRTLEQAVKGLNESKKLSKPSN

>IMP-44
MKKLFVLCIFLFCSITAAGASLPDLKIEKLEEGVYVHTSFEEVNGWGVFSKHGLVVLVNTDAYLIDTPSTAKDTEKLVNWFVERGYKIKGSISSHFHSDSTGGIEWLN
SQSIPTYASVLTNELLKKDGKVQAKNSFSGVSYWLVKNKIEVFYPGPGHTQDNVVVWLPKNKILFGGCFVKPYGLGNLDDANVEAWPHSAEKLISKYGNAKLVVP
SHSDIGDASLLKLTWEQAVKGLNESKKSNTVH

>IMP-45
MSKLFVFFMFLFCSITAAGESLPDLKIEKLDEGVYVHTSFEEVNGWGVIPKHGLVVLVNTDAYLIDTPFTAKDTENLVNWFVERGYRIKGSISSHFHSDSTGGIEWLN
SQSIPTYASELTNELLKKDGKVQAKYSFSGVSYWLVKKKIEVFYPGPGHAPDNVVVWLPENRVLFGGCFVKPYGLGNLGDANLEAWPKSAKLLMSKYSKAKLVVP
GHSDIGDSSLLKLTWEQTVKGFNESKKSTTAH

>IMP-46
MKKLFVLCVFLLCNIATAEDSLPDLKIDKLEEGVYVHTSFEEVKGWNVVTKHGLVVLVKNDAYLIDTPITVKDTEKLVNWLVERGYKIKGSISTHFHDDSSAGIEWL
NSQSIPTYASRLTNELRKKGGKPQATNSFDGVSYSLIKNKIEVFYPGPGHTQDNVVVWLPEKKILFGGCFVKPDGLGYLGDANLEAWPKSAKILMSKYAKAKLVVSS
HSEIGDTSLLKRTWEQAVKGLNESKKP

>IMP-48
MKKLFVLCVFFFCNIAVAEESLPDLKIEKLEEGVYVHTSFEEVKGWSVVTKHGLVVLVKNDAYLIDTPTTAKDTEKLVNWFVERGYKIKGSISTHFHGDSTAGIEWL
NSQSIPTYASELTNELLKKDNKVQAKHSFNGVSYSLIKNKIEVFYPGPGHTQDNVVVWLPEKKILFGGCFVKPDGLGYLGDANLEAWPKSAKILMSKYGKAKLVVS
SHSDIGDVSLLKRTWEQAVKGLNESKKSSQPSD

>IMP-49
MKKLFVLCVFFLCNIAAADDSLPDLKIEKLEKGVYVHTSFEEVKGWGVFTKHGLVVLVKNDAYLIDTPITAKDTEKLVNWFIEHGYRIKGSISTHFHGDSTAGIEWL
NSQSISTYASELTNELLKKDNKVQATNSFSGVSYSLIKNKIEVFYPGPGHTQDNVVVWLPEKKILFGGCFVKPDGLGNLGDANLEAWPKSAKILMSKYGKAKLVVSS
HSEIGNASLLQRTWEQAVKGLNESKKPLQPSS

>IMP-51
MKKLSVFFMFLFCSIAASGEALPDLKIEKLDEGVYVHTSFEEVNGWGVVPKHGLVVLVNTDAYLIDTPFTAKDTEKLVTWFVERGYKIKGSISSHFHSDSTGGIEWL
NSQSIPTYASELTNELLKKDGKVQAKNSFSGASYWLVKKKIEIFYPGPGHTPDNVVVWLPEHRVLFGGCFVKPYGLGNLGDANLEAWPKSAKLLVSKYGKAKLVVP
GHSEVGDASLLKRTLEQAVKGLNESKKLSKPSN

>IMP-52
MSKLSVFFIFLFCSIATAAESLPDLKIEKLDEGVYVHTSFEEVNGWGVVPKHGLVVLVNAEAYIIDTPFTAKDTEKLGTWFVERGYKIKGSISSHFHSDSTGGIEWLNS
RSIPTYASELTNELLKKDGKVQATNSFSGVNYWLVKNKIEVFYPGPGHTPDNVVVWLPERKILFGGCFIKPYGLGNLGDANIEAWPKSAKLLKSKYGKAKLVVPSHS
EVGDASLLKLTLEQAVKGLNESKKPSKPSN

>IMP-53
MSKLFVFFMFLFCSITAAGESLPDLKIEKLDEGVYVHTSFEEVNGWGVIPKHGLVVLVNTDAYLIDTPFTAKDTENLVNWFVERGYRIKGSISSHFHSDSTGGIEWLN
SQSIPTYASELTNELLKKDGKVQAKYSFSGVSYWLVKKKIEVFYPGSGHAPDNVVVWLPENRVLFGGCFVKPYGLGNLGDANLEAWPKSAKLLMSKYSKAKLVVP
GHSDIGDSSLLKLTWEQTVKGFNESKKSTTAH

>IMP-54
MKKLFVLCVFFFCNIAVAEESLPDLKIEKLEEGVYVHTSFEEVKGWSVVTKHGLVVLVKNDAYLIDTPVTAKDTEKLVNWFVERGYKIKGSISTHFHGDSTAGIEWL
NSQSIPTYASELTNELLKKDNKVQAKHSFNGVSYSLIKNKIEVFYPGPGHTQDNVVVWLPEKKILFGGCFVKPDGLGYLGDANLEAWPKSAKILMSKYGKAKLVVS
SHSDIGDVSLLKRTWEQAVKGLNESKKSSQPSD

>IMP-55
MSKLSVFFIFLICSIATAAESLPDLKIEKLEEGVYVHTSFKEVNGWGVVPKHGLVVLVNAEAYLIDTPFTAKDTEKLVTWFVERGYKIKGSISSHFHSDSTGGIEWLNS
RSIPTYASELTNELLKKDGKVQATNSFSGVNYWLVKNKIEVFYPGPGHTPDNVVVWLPERKIFFGGCFIKPYGLGKLGDANIEAWPKSAKLLKSKYGKAKLVVPSHS
EVGDASLLKLTLEQAVKGLNESKKPSKPSN

>IMP-56
MKKLFVLCVFFLCNIAAADDSLPDLKIEKLEKGVYVHTSFEEVKGWGVVTKHGLVVLVKNDAYLIDTPITAKDTEKLVNWFIEHGYRIKGSISTHFHGDSTAGIEWL
NSQSISTYASELTNELLKKDNKVQATNSFSGVSYSLIKNKIEVFYPGPGHTQDNVVVWLPEKKILFGGCFVKPDGLGNLGDANLEAWPKSAKILMSKYGKAKLVVS
GHSEIGNASLLQRTWEQAVKGLNESKKPLQPSS

>IMP-58

MKKLFVLCVFLFCSITAAGESLPDLKIEKLEEGVYVHTSFEEVNGWGVFSKHGLVILVNTDAYLIDTPFTAKDTEKLVTWFVERGYKIKGSISSHFHSDSTGGIEWLNS
QSIPTYASELTNDLLKQNGKVQAKNSFSGVSYWLVKNKIEVFYPGPGHTQDNVVVWLPEKKILFGGCFVKPYGLGNLDDANVVAWPHSAEILMSRYGNAKLVVPS
HSDIGDASLLKLTWEQAVKGLKESKKPSEPSN

>IMP-59
MSKLSVFFIFLFCSIATAAEPLPDLKIEKLDEGVYVHTSFEEVNGWGVVPKHGLVVLVDAEAYLIDTPFTAKDTEKLVTWFVERGYKIKGSISSHFHSDSTGGIEWLNS
QSIPTYASELTNELLKKDGKVQAKNSFGGVNYWLVKNKIEVFYPGPGHTPDNLVVWLPERKILFGGCFIKPYGLGYLGDANLEAWPKSAKLLISKYGKAKLVVPSH
SEAGDASLLKLTLEQAVKGLNESKKPSKLSN

>IMP-60
MSKLSVFFIFLFCSIATAAESLPDLKIEKLDEGVYVHTSFEEVNGWGVVPKHGLVVLVNAEAYLIDTPFTAKDTEKLVTWFVERGYKIKGSISSHFHSDSTGGIEWLNS
RSIPTYASELTNELLKKDGKVQATNSFSGVNYWLVKNKIEVFYPGPGHTPDNVVVWLPKRKILFGGCFIKPYGLGNLGDANIEAWPKSAKLLKSKYGKAKLVVPSH
SEVGDASLLKLTLEQAVKGLNESKKPSKPSN

>IMP-61
MSKLSVFFIFLFCSIATAAESLPDLKIEKLDEGVYVHTSFEEVNGWGVVPKHGLVVLVNAEAYLIDTPFTAKDTEKLVTWFVERGYKIKGSISSHFHSDSTGGIEWLIS
RSIPTYASELTNELLKKDGKVQATNSFSGVNYWLVKNKIEVFYPGPGHTPDNVVVWLPERKILFGGCFIKPYGLGNLGDANIEAWPKSAKLLKSKYGKAKLVVPSHS
EVGDASLLKLTLEQAVKGLNESKKPSKPSN

>IMP-62
MNKLSVFFMFMFCSITAAGESLPDLKIEKLDEGVYVHTSFEEVNGWGVVPKHGLVVLVNTEAYLIDTPFTAKDTEKLVTWFVERGYKIKGSISSHFHSDSTGGIEWL
NSQSIPTYASELTNELLKKDGKVQAKNSFSGGSYWLVNNKIEVFYPGPGHTPDNVVVWLPENRVLFGGCFVKPYGLGNLGDANLEAWPKSAKILMSKYGKAKLVV
SGHSETGNASLLKLTWEQAVKGLKESKKPSLPSN

>IMP-63
MKKLFVLCIFLFLSITASGEVLPDLKIEKLEEGVYLHTSFEEVSGWGVVTKHGLVVLVNNDAYLIDTPFTNKDTEKLVAWFVGRGFTIKGSVSSHFHSDSTGGIEWLN

SQSIPTYASELTNELLKKNGKVQATNSFSGVSYWLVKNKIEIFYPGPGHTQDNVVVWLPENKILFGGCFVKPDGLGNLDDANLKAWPKSAKILMSKYGKAKLVVSS
HSEIGNASLLKLTWEQAVKGLKESKKPLLPSN

>IMP-64
MKKLFVLCVFVFCSITVAAETLPNLRVEKLEEGVYVHTSYEEVKGWGVVTKHGLVVLIGADAYLIDTPFTAKDTEKLVNWFVERGYKIKGTVSSHFHSDSTGGIEW
LNSQSIPTYASELTNELLKKDGKVQAKNSFDGVSYWLAKDKIEVFYPGPGHTQDNVVVWLPEKEILFGGCFVKPHGLGNLGDANLEAWPESAKILMEKYGKAKLV
VSGHSETGDATHLKRTWEQAVKGLKESKKTLQPSN

>IMP-65
MKKLFVLCVFFFCNIAVAEESLPDLKIEKLEEGVYVHTSFEEVKGWSVVTKHGLVVLVKNDAYLIDTPITAKDTEKLVNWFVERGYKIKGSISTHFHGDSAAGIEWL
NSQSIPTYASELTNELLKKDNKVQAKHSFNGVSYSLIKNKIEVFYPGPGHTQDNVVVWLPEKKILFGGCFVKPDGLGYLGDANLEAWPKSAKILMSKYGKAKLVVS
SHSDIGDVSLLKRTWEQAVKGLNESKKSSQPSD

>IMP-66
MSKLSVFFIFLFCSIATAAESLPDLKIEKLDEGVYVHTSFEEVNGWGFVPKHGLVVLVNAEAYLIDTPFTAKDTEKLVTWFVERGYKIKGSISSHFHSDSTGGIEWLNS
RSIPTYASELTNELLKKDGKVQATNSFSGVNYWLVKNKIEVFYPGPGHTPDNVVVWLPERKILFGGCFIKPYGLGNLGDANIEAWPKSAKLLKSKYGKAKLVVPSHS
EVGDASLLKLTLEQAVKGLNESKKPSKPSN

>IMP-67
MKKLFVLCVFVFCSITVAGETLPNLRVEKLEEGVYVHTSYEEVKGWGVVTKHGLVVLIGADAYLIDTPFTAKDTEKLVNWFVERGYKIKGTVSSHFHSDSTGGIEW
LNSQSIPTYASELTNELLKKDGKVQAKNSFDGVSYWLAKDKIEVFYPGPGHTQDNVVVWLPEKEILFGGCFVKPHGLGNLGDANLEAWPESAKILMEKYGKAKLV
VSGHSETGDSTHLKRTWEQAVKGLKESKKTLQPSN

>IMP-68
MKKLFVLCIFLFCSITAAGASLPDLKIEKLEEGVYVHTSFEEVNGWGVVSKHGLVVLVNTDAYLIDTPFTAKDTEKLVNWFVERGYKIKGSISSHFHSDSTGGIEWLN
SQSIPTYASVLTNELLKKDGKVQAKNSFSGVSYWLVKNKIEVFYPGPGHTQDNVVVWLPKNKILFGGCFVKPYGLGNLDDANVEAWPHSAEKLISKYGNAKLVVP

GHSDIGDASLLKLTWEQAVKGLNESKKSNTVH

>IMP-69
MKKLFVLCVCFLCSITAAGATLPDLKIEKLEEGVYVHTSFEEVNGWGVVSKHGLVVLVNTDAYLIDTPFTATDTEKLVNWFVERGYKIKGTISSHFHSDSTGGIEWL
NSQSIPTYASELTNELLKKDGKVQAKNSFSGVSYWLVKNKIEVFYPGPGHTQDNVVVWLPEKKILFGGCFVKPDGLGNLGDANLEAWPKSAKILMSKYGKAKLVV
SSHSEIGDASLLKRTWEQAVKGLNESKKPSQPSN

>IMP-70
MSKLSVFFIFLFCSIATAAESLPDLKIEKLDEGVYVHTSFEEVNGWGVVPKHGLVVLVNAEAYLIDTPFTAKDTEKLVTWFVERGYKIKGSISSHFHSDSTGGIEWLNS
RSIPTYASELTNELLKKDGKVQATNSFSGVNYWLVKNKIEVFYPGPGHTPDNVVVWLPERKILFGGCFIKPYGLGNLGDANIEAWPKSAKLLKSKYGKAKLVVPSHS
EVGDASLLKLTIEQAVKGLNESKKPSKPSN

>IMP-71
MKKLFVLCVFFLCNIAAADDSLPDLKIEKLEKGVYVHTSFEEVKGWGVFAKHGLVVLVKNDAYLIDTPITAKDTEKLVNWFIEHGYRIKGSISTHFHGDSTAGIEWL
NSQSISTYASELTNELLKKDNKVQATNSFSGVSYSLIKNKIEVFYPGPGHTQDNVVVWLPEKKILFGGCFVKPDGLGNLGDANLEAWPKSAKILMSKYGKAKLVVSS
HSEIGNASLLQRTWEQAVKGLNESKKPLQPSS

>IMP-73
MKKLSVFFMFLFCSIAASGEALPDLKIEKLDEGVYVHTSFEEVNGWGVAPKHGLVVLVNTDAYLIDTPFTAKDTEKLVTWFVERGYKIKGSISSHFHSDSTGGIEWL
NSQSIPTYASELTNELLKKDGKVQAKNSFSGASYWLVKKKIEIFYPGPGHTPDNVVVWLPEHRVLFGGCFVKPYGLGNLGDANLEAWPKSAKLLVSKYGKAKLVVP
SHSEVGDASLLKRTLEQAVKGLNESKKLSKPSN

>IMP-74
MKKLFVLCIFLFCSITAAGESLPDLKIEKLEDGVYVHTSFEEVNGWGVFTKHGLVFLVNTDAYLIDTPFAAKDTEKLVNWFVERGYKIKGSISSHFHSDSSGGIEWLN
SQSIPTYASELTNELLKKNGKVQAKNSFSGVSYWLLKNKIEIFYPGPGHTQDNVVVWLPEKKILFGGCFVKPYGLGNLDDANVEAWPHSAEILMSRYGNAKLVVPS
HSDVGDASLLKLTWEQAVKGLKESKKPSQPSN

>IMP-75
MKKLFVLCVFFLCNIAAADDSLPDLKIEKLEKGVYVHTSFEEVKGWGVFAKHGLVVLVKNDAYLIDTPITAKDTEKLVNWFIEHGYRIKGSISTHFHGDSTAGIEWL
NSQSISTYASELTNELLKKDNKVQATNSFSGVSYSLIKNKIEVFYPGPGHTQDNVVVWLPEKKILFGGCFVKPDGLGNLGDANLEAWPKSAKILMSKYGKAKLVVPS
HSEIGNASLLQRTWEQAVKGLNESKKPLQPSS

>IMP-76
MSKLSVFFIFLFCSIATAAESLPDLKIEKLDEGVYVHTSFEEVNGWGVAPKHGLVVLVNAEAYLIDTPFTAKDTEKLVTWFVERGYKIKGSISSHFHSDSTGGIEWLNS
RSIPTYASELTNELLKKDGKVQATNSFSGVNYWLVKNKIEVFYPGPGHTPDNVVVWLPERKILFGGCFIKPYGLGNLGDANIEAWPKSAKLLKSKYGKAKLVVPSHS
EVGDASLLKLTLEQAVKGLNESKKPSKPSN

>IMP-77
MSKLSVFFIFLFCSIATAAESLPDLKIEKLDEGVYVHTSFEEVNGWGVFPKHGLVVLVNAEAYLIDTPFTAKDTEKLVTWFVERGYKIKGSISSHFHSDSTGGIEWLNS
RSIPTYASELTNELLKKDGKVQATNSFSGVNYWLVKNKIEVFYPGPGHTPDNVVVWLPERKILFGGCFIKPYGLGNLGDANIEAWPKSAKSLKSKYGKAKLVVPSHS
EVGDASLLKLTLEQAVKGLNESKKPSKPSN

>IMP-78
MSKLSVFFIFLFCSIATAAESLPDLKIEKLDEGVYVHTSFEEVNGWGVFPKHGLVVLVNAEAYLIDTPFTAKDTEKLVTWFVERGYKIKGSISSHFHSDSTGGIEWLNS
RSIPTYASELTNELLKKDGKVQATNSFSGVNYWLVKNKIEVFYPGPGHTPDNVVVWLPERKILFGGCFIKPYGLGNLGDANIEAWPKSAKLLKSKYGKAKLVVPGH
SEVGDASLLKLTLEQAVKGLNESKKPSKPSN

>IMP-79
MSKLSVFFIFLFCSIATAAESLPDLKIEKLDEGVYVHTSFEEVNGWGVVPKHGLVVLVNAEAYLIDTPFTAKDTEKLVTWFVERGYKIKGSISSHFHSDSTGGIEWLNS
RSIPTYASELTNELLKKDGKVQATNSFSGVNYWLVKNKIEVFYPGPGHTPDNVVVWLPERKILFGGCFIKPYGLGNLGDANIEAWPKSARLLKSKYGKAKLVVPSHS
EVGDASLLKLTLEQAVKGLNESKKPSKPSN

>IMP-80
MSKLSVFFIFLFCSIATAAESLPDLKIEKLDEGVYVHTSFEEVNGWGVFPKHGLVVLVNAEAYLIDTPFTAKDTEKLVTWFVERGYKIKGSISSHFHSDSTGGIEWLNS
RSIPTYASELTNELLKKDGKVQATNSFSGVNYWLVKNKIEVFYPGPGHTPDNVVVWLPERKILFGGCFIKPYGLGNLGDANIEAWPKSAKLLKSKYGKAKLVVPAHS
EVGDASLLKLTLEQAVKGLNESKKPSKPSN

>IMP-81
MSKLFVFCMFLFCSITAAGESLPDLKIEKLDEGVYVHTSFEEVNGWGVVPKHGLVVLVNTDAYLIDTPFTAKDTEKLVTWFVERGYKIKGSVSSHFHSDSTGGIEWL
NSQSIPTYASELTNELLKKDGKVQAKNSFSGVSYWLVKNKVEIFYPGPGHTPDNVVVWLPENRVLFGGCFVKPYGLGNLGDANLEAWPKSAKLLMSKYGKAKLV
VPSHSEVGDASLLKLTLVQAVKGLNESKKPSKPSN

>IMP-82
MSKLFVFFMFLFCSITAAGESLPDLKIEKLDEGVYVHTSFEEVNGWGVVPKHGLVVLVNTDAYLIDTPFTAKDTENLVNWFVERGYKIKGSISSHFHSDSTGGIEWLN
SQSIPTYASELTNELLKKDGKVQAKNSFSGVSYWLVKKKIEVFYPGPGHAPDNVVVWLPENRVLFGGCFVKPYGLGNLGDANLEAWPKSAKLLMSKYSKAKLVVP
SHSDIGDSSLLKLTWEQTVKGFNERKKSTTAH

>IMP-83
MKKLFVLCVFFLCNIAAADDSLPDLKIEKLEKGVYVHTSFEEVKGWGVVTKHGLVVLVKNDAYLIDTPVTAKDTEKLVNWFIEHGYRIKGSISTHFHGDSTAGIEWL
NSQSISTYASELTNELLKKDNKVQATNSFSGVSYSLIKNKIEVFYPGPGHTQDNVVVWLPEKKILFGGCFVKPDGLGNLGDANLEAWPKSAKILMSKYGKAKLVVSS
HSEIGNASLLQRTWEQAVKGLNESKKPLQPSS

>IMP-84
MKKLFVLCVCFFCSITAAGAALPDLKIEKLEEGVFVHTSFEEVNGWGVFTKHGLVVLVNTDAYLIDTPFTATDTEKLVNWFVERGYEIKGTISSHFHSDSTGGIEWLN
SQSIPTYASELTNELLKKSGKVQAKYSFSEVSYWLVKNKIEVFYPGPGHTQDNLVVWLPESKILFGGCFIKPHGLGNLGDANLEAWPKSAKILMSKYGKAKLVVSSH
SEKGDASLMKRTWEQALKGLKESKKTSSPSN

>IMP-85

MSKLFVFFMFLFCSITAAAESLPDLKIEKLDEGVYVHTSFEEVNGWGVVPKHGLVVLVNTEAYLIDTPFTAKDTEKLVTWFVERGYKIKGSISSHFHSDSTGGIEWLN
SQSIPTYASELTNELLKKDGKVQAKNSFSGASYWLVKKKIEVFYPGPGHTPDNVVVWLPENRVLFGGCFVKPYGLGNLGDANVEAWPKSAKLLMSKYGKAKLVVP
SHSEVGDASLLKRTLEQAVKGLNESKKPSKPGN

>IMP-86
MKKLFVLCVFFFCNIAVAEESLPDLKIEKLEEGVYVHTSFEEAKGWGVVTKHGLVVLVKNDAYLIDTPVTAKDTEKLVNWFVERGYKIKGSISTHFHGDSTAGIEWL
NSQSIPTYASELTNELLKKDNKVQAKHSFNGVSYSLIKNKIEVFYPGPGHTQDNVVVWLPEKKILFGGCFVKPDGLGYLGDANLEAWPKSAKILMSKYGKAKLVVS
SHSDIGDVSLLKRTWEQAVKGLNESKKSSQPSD

>IMP-87
MKKLFVLCVFFFCNIAVAEESLPDLKIEKLEEGVYVHTSFEEAKGWGVVTKHGLVVLVKNDAYLIDTPITAKDTEKLVNWFVERGYKIKGSISTHFHGDSTAGIEWL
NSQSIPTYASELTNELLKKDNKVQAKHSFNGVSYSLIKNKIEVFYPGPGHTQDNVVVWLPEKKILFGGCFVKPDGLGYLGDANLEAWPKSAKILMSKYGKAKLVVS
SHSDIGDVSLLKRTWEQAVKGLNESKKSSQPSD

>IMP-88
MSKLSVFFIFLFCSIATAAESLPDLKIEKLDEGVYVHTSFEEVNGWGVFPKHGLVVLVNAEAYLIDTPFTAKDTEKLVTWFVERGYKIKGSISSHFHSDSTGGIEWLNS
RSIPTYASELTNELLKKHGKVQATNSFSGVNYWLVKNKIEVFYPGPGHTPDNVVVWLPERKILFGGCFIKPYGLGNLGDANIEAWPKSAKLLKSKYGKAKLVVPSHS
EVGDASLLKLTLEQAVKGLNESKKPSKPSN

>IMP-89
MSKLSVFFIFLFCNIATAAEPLPDLKIEKLDEGVYVHTSFEEVNGWGVFPKHGLVVLVDAEAYLIDTPFTAKDTEKLVTWFVERGYKIKGSISSHFHSDSTGGIEWLNS
QSIPTYASELTNELLKKDGKVQAKNSFGGVNYWLVKNKIEVFYPGPGHTPDNLVVWLPERKILFGGCFIKPYGLGNLGDANLEAWPKSAKLLISKYGKAKLVVPSH
SEAGDASLLKLTLEQAVKGLNESKKPSKLSN

>IMP-90
MKKLFVLCIFLFLSITASGEVLPDLKIEKLEEGVYLHTSFEEVSGWGVVTKHGLVVLVNNDAYLIDTPSTTKDTEKLVAWFVERGFTIKGSVSSHFHSDSTGGIEWLNS

QSIPTYASELTNELLKKNGKVQATNSFSGVSYWLVKNKIEIFYPGPGHTQDNVVVWLPENKILFGGCFVKPDGLGNLDDANLKAWPKSAKILMSKYGKAKLVVSSH
SEIGNASLLKLTWEQAVKGLKESKQPLLPSN

>IMP-91
MKKLFVLCVFFFCNIAVAEESLPDLKIEKLEEGVYVHTSFEEAKGWSVVTKHGLVVLVKNDAYLIDTPITAKDTEKLVNWFVERGYKIKGSISTHFHGDSTAGIEWLN
SQSIPTYASELTNELLKKDNKVQAKHSFNGVSYSLIKNKIEVFYPGPGHTQDNVVVWLPEKKILFGGCFVKPDGLGYLGDANLEAWPKSAKILMSKYGKAKLVVSS
HSDIGDVSLLKRTWEQAVKGLNESKKSSQPSD

>IMP-92
MKKIFVLFVFLFCSITAAGESLPDIKIEKLDEDVYVHTSFEKITGWGVITKHGLVVLVNTDAYIIDTPFTAKDTEKLVRWFVGRGYKIKGSISSHFHSDSAGGIEWLNSQ
SIPTYASKLTNELLKKNGNAQAVNSFSGVSYWLVKHKIEVFYPGPGHTQDNVVVWLPEKKILFGGCFIKPDGLGYLGDANLEAWPKSAETLMSKYGNAKLVVSSHS
EIGGASLLKRTWEQAVKGLKESKNHHSPK

>IMP-93
MKKLFVLCIFLFCSITAAGESLPDLKIEKLEDGVYVHTSFEEVNGWGVFAKHGLVFLVNTDAYLIDTPFAAKDTEKLVNWFVERGYKIKGSISSHFHSDSSGGIEWLN
SQSIPTYASELTNELLKKNGKVQAKNSFSGVSYWLLKNKIEIFYPGPGHTQDNVVVWLPEKKILFGGCFVKPYGLGNLDDANVEAWPHSAEILMSRYGNAKLVVPS
HSDVGDASLLKLTWEQAVKGLKESKKPSQPSN

>IMP-94
MSKLFIFFMFLFCSITAAAESLPDLKIERLDEGVYVHTSFEEVNGWGVVPKHGLVVLVNTEAYLIDTPFTAKDTEKLVTWFVGRGYKIKGSISSHFHSDSTGGIEWLNS
QSIPTYASELTNELLKKDGKVQAKNSFGGVSYWLVKNKIEVFYPGPGHTPDNVVVWLPENRVLFGGCFVKPYGLGNLGDANLEAWPKSAKLLMSKYGKAKLVVP
SHSEVGDASLLKRTLEHAVKGLNESKKPSKPSN

>IMP-95
MKKLFVLCVCFFCSITAAGAALPDLKIEKLEEGVFVHTSYEEVKGWGVVTKHGLVVLIGADAYLIDTPFTAKDTEKLVNWFVERGYKIKGTVSSHFHSDSTGGIEWL
NSQSIPTYASELTNELLKKDGKVQAKNSFDGVSYWLAKDKIEVFYPGPGHTQDNVVVWLPEKEILFGGCFVKPHGLGNLGDANLEAWPESAKILMEKYGKAKLVV

SGHSETGDATHLKRTWEQAVKGLKESKKTLQPSN

>IMP-96
MKKLFVLCVCFLCSITAAGAALPDLKIEKLEEGVYVHTSFEEVNGWGVVSKHGLVVLVNTDAYLIDTPFTATDTEKLVNWFVERGYKIKGTISSHFHSDSTGGIEWL
NSQSIPTYASELTNELLKKDGKVQAKNSFSGVSYWLVKNKIEVFYPGPGHTQDNVVVWLPEKKILFGGCFVKPDGLGNLGDANLEAWPKSAKILMSKYGKAKLVV
SGHSEIGDASLLKRTWEQAVKGLNESKKPSQPSN

Table S3. Pairwise comparison of IMP sequences using BLASTP								
**Identity (%)**	**IMP-52**	**IMP-42**	**IMP-79**	**IMP-70**	**IMP-66**	**IMP-60**	**IMP-30**	**IMP-34**
**IMP-52**	100	99	99	99	99	99	99	99
**IMP-42**	99	100	99	99	99	99	99	99
**IMP-79**	99	99	100	99	99	99	99	99
**IMP-70**	99	99	99	100	99	99	99	99
**IMP-66**	99	99	99	99	100	99	99	99
**IMP-60**	99	99	99	99	99	100	99	99
**IMP-30**	99	99	99	99	99	99	100	99
**IMP-34**	99	99	99	99	99	99	99	100
**IMP-1**	99	99	99	99	99	99	99	99
**IMP-61**	99	99	99	99	99	99	99	99
**IMP-80**	98	99	99	99	99	99	99	99
**IMP-78**	98	99	99	99	99	99	99	99
**IMP-6**	99	99	99	99	99	99	99	99
**IMP-3**	98	99	99	99	99	99	99	99
**IMP-25**	98	99	99	99	99	99	99	99
**IMP-76**	99	99	99	99	99	99	99	99
**IMP-40**	98	99	99	99	99	99	99	99
**IMP-77**	98	99	99	99	99	99	99	99
**IMP-10**	99	99	99	99	99	99	99	99
**IMP-88**	98	99	99	99	99	99	99	99
**IMP-55**	97	98	98	98	98	98	98	98
**IMP-38**	95	95	95	95	95	95	95	95
**IMP-4**	95	96	96	96	96	96	96	96
**IMP-59**	95	95	95	95	95	95	95	95
**IMP-26**	95	95	95	95	95	95	95	95
**IMP-89**	94	95	95	95	95	95	95	95
**IMP-5**	92	93	93	93	93	93	93	93
**IMP-85**	92	92	92	92	92	92	92	92
**IMP-28**	91	92	92	92	92	92	92	92
**IMP-94**	91	91	91	91	91	91	91	91
**IMP-81**	91	91	91	91	91	91	91	91
**IMP-51**	90	90	90	90	90	90	90	90
**IMP-43**	90	90	90	90	90	90	90	90
**IMP-7**	90	91	91	91	91	91	91	91
**IMP-73**	90	90	90	90	90	90	90	90
**IMP-15**	89	89	89	90	89	89	89	89
**IMP-62**	89	89	89	89	89	89	89	89
**IMP-29**	88	88	89	89	88	88	88	88
**IMP-82**	87	87	87	88	87	87	87	87
**IMP-9**	86	86	86	87	86	86	86	86
**IMP-45**	86	86	86	86	86	86	86	86
**IMP-53**	85	86	86	86	86	86	86	86
**IMP-21**	86	86	87	87	86	87	86	86
**IMP-11**	86	87	87	87	87	88	87	87
**IMP-68**	86	86	87	87	86	87	86	86
**IMP-41**	86	86	87	87	86	87	86	86
**IMP-44**	85	86	86	86	86	87	86	86
**IMP-22**	85	85	85	85	85	85	85	85
**IMP-58**	84	85	85	85	85	85	85	85
**IMP-16**	84	85	85	85	85	85	85	85

IMP-74	84	84	85	85	84	84	84	84
IMP-93	84	84	85	85	84	84	84	84
IMP-2	85	85	85	85	85	85	85	85
IMP-19	85	85	85	85	85	85	85	85
IMP-8	85	85	85	86	85	85	85	85
IMP-24	85	85	85	85	85	85	85	85
IMP-96	85	85	85	85	85	85	85	85
IMP-69	85	85	85	86	85	85	85	85
IMP-20	84	85	85	85	85	85	85	85
IMP-23	85	85	85	85	85	85	85	85
IMP-39	83	84	84	84	84	84	84	84
IMP-33	83	83	83	83	83	83	83	83
IMP-17	82	82	82	83	82	82	82	82
IMP-13	82	82	82	83	82	82	82	82
IMP-37	81	81	81	82	81	81	81	81
IMP-84	81	82	82	82	82	82	82	82
IMP-90	82	82	82	83	82	82	82	82
IMP-12	82	82	82	83	82	82	82	82
IMP-63	82	83	83	83	83	83	83	83
IMP-48	80	81	81	81	81	81	81	81
IMP-32	80	81	81	81	81	81	81	81
IMP-14	80	81	81	81	81	81	81	81
IMP-65	80	80	80	81	80	80	80	80
IMP-54	80	81	81	81	81	81	81	81
IMP-91	80	80	80	81	80	80	80	80
IMP-86	80	81	81	81	81	81	81	81
IMP-87	80	81	81	81	81	81	81	81
IMP-83	80	80	80	80	80	80	80	80
IMP-18	80	80	80	80	80	80	80	80
IMP-56	79	80	80	80	80	80	80	80
IMP-49	79	80	80	80	80	80	80	80
IMP-71	79	80	80	80	80	80	80	80
IMP-75	80	80	80	80	80	80	80	80
IMP-46	79	80	80	80	80	80	80	80
IMP-35	82	82	82	82	82	82	82	82
IMP-31	80	80	80	80	80	80	80	80
IMP-92	80	80	80	80	80	80	80	80
IMP-95	79	79	79	80	79	79	79	79
IMP-27	79	79	79	80	79	79	79	79
IMP-64	79	80	80	80	80	80	80	80
IMP-67	78	79	79	79	79	79	79	79

Table S3. (continued)								
**Identity (%)**	**IMP-1**	**IMP-61**	**IMP-80**	**IMP-78**	**IMP-6**	**IMP-3**	**IMP-25**	**IMP-76**
IMP-52	99	99	98	98	99	98	98	99
IMP-42	99	99	99	99	99	99	99	99
IMP-79	99	99	99	99	99	99	99	99
IMP-70	99	99	99	99	99	99	99	99
IMP-66	99	99	99	99	99	99	99	99
IMP-60	99	99	99	99	99	99	99	99
IMP-30	99	99	99	99	99	99	99	99
IMP-34	99	99	99	99	99	99	99	99
IMP-1	100	99	99	99	99	99	99	99
IMP-61	99	100	99	99	99	99	99	99
IMP-80	99	99	100	99	99	99	99	99
IMP-78	99	99	99	100	99	99	99	99
IMP-6	99	99	99	99	100	99	99	99
IMP-3	99	99	99	99	99	100	99	99
IMP-25	99	99	99	99	99	99	100	99
IMP-76	99	99	99	99	99	99	99	100
IMP-40	99	99	99	99	99	98	98	99
IMP-77	99	99	99	99	99	98	98	99
IMP-10	99	99	99	99	99	99	99	99
IMP-88	99	99	99	99	99	98	98	99
IMP-55	98	98	97	97	98	97	97	98
IMP-38	96	95	95	96	96	96	96	95
IMP-4	96	96	95	95	96	95	95	96
IMP-59	96	95	95	95	95	95	95	95
IMP-26	96	95	96	96	95	95	95	96
IMP-89	95	95	95	95	95	94	94	95
IMP-5	93	93	92	92	93	92	92	93
IMP-85	93	92	92	92	92	92	92	92
IMP-28	92	92	91	91	92	91	91	92
IMP-94	92	91	91	91	91	91	91	91
IMP-81	91	91	91	91	91	91	91	91
IMP-51	91	90	90	91	91	91	91	90
IMP-43	91	90	91	91	90	90	90	91
IMP-7	91	91	90	90	91	90	90	91
IMP-73	91	90	90	90	90	90	90	91
IMP-15	90	89	89	89	89	89	89	89
IMP-62	89	89	89	89	90	89	89	89
IMP-29	89	88	88	88	88	88	88	88
IMP-82	88	87	87	87	87	87	87	87
IMP-9	87	86	86	86	86	86	86	87
IMP-45	86	86	86	87	87	86	86	86
IMP-53	86	86	86	86	86	86	86	86
IMP-21	87	86	86	86	86	86	86	87
IMP-11	87	87	86	86	87	86	87	87
IMP-68	87	86	86	87	87	87	87	86
IMP-41	87	86	87	87	86	86	86	87
IMP-44	86	86	86	86	86	85	86	86
IMP-22	85	85	85	85	85	85	85	85
IMP-58	85	85	85	85	85	84	85	85
IMP-16	85	85	84	84	85	84	85	85

IMP-74	85	84	85	85	84	84	84	85
IMP-93	85	84	85	85	84	84	84	85
IMP-2	85	85	85	85	85	85	85	85
IMP-19	85	85	85	85	85	85	85	85
IMP-8	86	85	85	85	85	85	85	85
IMP-24	85	85	85	85	85	85	85	85
IMP-96	85	85	85	85	86	85	85	85
IMP-69	86	85	85	85	85	85	85	85
IMP-20	85	85	85	85	85	84	84	85
IMP-23	85	85	85	85	85	85	85	85
IMP-39	84	84	83	83	84	83	83	84
IMP-33	83	83	83	83	83	83	83	83
IMP-17	83	82	82	82	82	82	82	82
IMP-13	83	82	82	82	82	82	82	82
IMP-37	82	81	81	81	81	81	81	81
IMP-84	82	82	82	82	82	81	81	82
IMP-90	83	82	82	82	82	82	82	82
IMP-12	83	82	82	83	83	83	83	82
IMP-63	83	83	82	82	83	82	83	83
IMP-48	81	81	80	80	81	80	80	81
IMP-32	81	81	80	80	81	80	80	81
IMP-14	81	81	80	80	81	80	80	81
IMP-65	81	80	80	80	80	80	80	80
IMP-54	81	81	80	80	81	80	80	81
IMP-91	81	80	80	80	80	80	80	80
IMP-86	81	81	80	80	81	80	80	81
IMP-87	81	81	80	80	81	80	80	81
IMP-83	80	80	80	80	80	80	80	80
IMP-18	80	80	80	80	80	80	80	80
IMP-56	80	80	80	80	80	80	80	80
IMP-49	80	80	80	80	80	79	79	80
IMP-71	80	80	80	80	80	79	79	80
IMP-75	80	80	80	80	80	80	80	80
IMP-46	80	80	79	79	80	79	79	80
IMP-35	82	82	82	82	82	81	81	82
IMP-31	80	80	80	80	80	80	80	80
IMP-92	80	80	80	80	80	80	80	80
IMP-95	80	79	79	80	80	80	80	79
IMP-27	80	79	79	80	80	80	80	79
IMP-64	80	80	80	80	80	80	80	80
IMP-67	79	79	79	79	80	79	79	79

Table S3. (continued)								
Identity (%)	IMP-40	IMP-77	IMP-10	IMP-88	IMP-55	IMP-38	IMP-4	IMP-59
IMP-52	98	98	99	98	97	95	95	95
IMP-42	99	99	99	99	98	95	96	95
IMP-79	99	99	99	99	98	95	96	95
IMP-70	99	99	99	99	98	95	96	95
IMP-66	99	99	99	99	98	95	96	95
IMP-60	99	99	99	99	98	95	96	95
IMP-30	99	99	99	99	98	95	96	95
IMP-34	99	99	99	99	98	95	96	95
IMP-1	99	99	99	99	98	96	96	96
IMP-61	99	99	99	99	98	95	96	95
IMP-80	99	99	99	99	97	95	95	95
IMP-78	99	99	99	99	97	96	95	95
IMP-6	99	99	99	99	98	96	96	95
IMP-3	98	98	99	98	97	96	95	95
IMP-25	98	98	99	98	97	96	95	95
IMP-76	99	99	99	99	98	95	96	95
IMP-40	100	99	99	99	97	95	95	95
IMP-77	99	100	99	99	97	95	95	95
IMP-10	99	99	100	99	98	95	96	95
IMP-88	99	99	99	100	97	95	95	95
IMP-55	97	97	98	97	100	93	94	94
IMP-38	95	95	95	95	93	100	99	99
IMP-4	95	95	96	95	94	99	100	99
IMP-59	95	95	95	95	94	99	99	100
IMP-26	96	96	96	96	93	99	99	99
IMP-89	95	95	96	95	93	99	99	99
IMP-5	92	92	93	92	91	91	91	91
IMP-85	92	92	92	92	91	91	91	91
IMP-28	91	91	92	91	90	91	92	91
IMP-94	91	91	91	91	90	91	91	91
IMP-81	91	91	91	91	89	90	90	90
IMP-51	90	90	90	90	89	90	90	89
IMP-43	91	91	91	91	89	89	90	89
IMP-7	90	90	91	90	89	90	90	90
IMP-73	90	90	91	90	89	89	90	89
IMP-15	89	89	89	89	88	89	89	89
IMP-62	89	89	89	89	87	89	89	88
IMP-29	88	88	88	88	87	87	87	87
IMP-82	87	87	87	87	86	87	87	87
IMP-9	86	86	87	86	85	86	86	86
IMP-45	86	86	86	86	84	86	86	86
IMP-53	86	86	86	86	84	86	86	85
IMP-21	86	87	87	86	85	84	85	84
IMP-11	86	87	87	86	86	85	85	85
IMP-68	86	86	86	86	85	85	85	84
IMP-41	87	87	87	87	85	84	85	84
IMP-44	87	87	87	86	85	84	84	84
IMP-22	85	85	85	85	84	84	84	84
IMP-58	85	85	85	85	84	83	84	83
IMP-16	84	85	85	85	84	83	83	83

IMP-74	85	85	85	85	83	83	83	83
IMP-93	85	85	85	85	83	83	83	83
IMP-2	85	85	85	85	85	85	85	85
IMP-19	85	85	85	85	85	85	85	85
IMP-8	85	85	85	85	85	85	85	85
IMP-24	85	85	85	85	85	85	85	85
IMP-96	85	85	85	85	85	85	85	85
IMP-69	85	85	85	85	85	85	85	85
IMP-20	85	85	85	85	84	84	85	84
IMP-23	85	86	86	85	85	85	85	85
IMP-39	83	84	84	83	83	83	84	83
IMP-33	83	83	83	83	82	83	83	83
IMP-17	82	82	82	82	81	83	83	83
IMP-13	82	82	82	82	81	83	83	83
IMP-37	81	81	81	81	80	82	83	82
IMP-84	82	83	83	83	81	82	83	82
IMP-90	83	82	82	82	81	81	81	81
IMP-12	82	82	82	82	81	82	81	81
IMP-63	82	83	83	83	82	81	82	81
IMP-48	81	81	81	80	80	81	81	82
IMP-32	81	81	81	80	80	81	81	82
IMP-14	81	81	81	80	80	81	81	82
IMP-65	80	80	80	80	80	80	81	81
IMP-54	81	81	81	80	80	81	81	82
IMP-91	80	80	80	80	80	80	81	81
IMP-86	81	81	81	80	80	81	81	82
IMP-87	81	81	81	80	80	81	81	82
IMP-83	80	80	80	80	80	80	80	80
IMP-18	80	80	80	80	80	80	80	80
IMP-56	80	80	80	79	79	80	80	80
IMP-49	80	80	80	80	79	80	80	80
IMP-71	80	80	80	80	79	80	80	80
IMP-75	81	81	81	80	80	80	80	80
IMP-46	80	80	80	80	80	79	80	80
IMP-35	82	82	82	82	80	80	81	81
IMP-31	80	80	80	80	79	80	81	81
IMP-92	80	80	80	80	79	80	81	81
IMP-95	79	79	79	79	78	80	80	80
IMP-27	79	79	79	79	78	80	80	80
IMP-64	79	80	80	79	79	81	80	80
IMP-67	78	79	79	78	78	80	80	79

Table S3. (continued)								
Identity (%)	IMP-26	IMP-89	IMP-5	IMP-85	IMP-28	IMP-94	IMP-81	IMP-51
IMP-52	95	94	92	92	91	91	91	90
IMP-42	95	95	93	92	92	91	91	90
IMP-79	95	95	93	92	92	91	91	90
IMP-70	95	95	93	92	92	91	91	90
IMP-66	95	95	93	92	92	91	91	90
IMP-60	95	95	93	92	92	91	91	90
IMP-30	95	95	93	92	92	91	91	90
IMP-34	95	95	93	92	92	91	91	90
IMP-1	96	95	93	93	92	92	91	91
IMP-61	95	95	93	92	92	91	91	90
IMP-80	96	95	92	92	91	91	91	90
IMP-78	96	95	92	92	91	91	91	91
IMP-6	95	95	93	92	92	91	91	91
IMP-3	95	94	92	92	91	91	91	91
IMP-25	95	94	92	92	91	91	91	91
IMP-76	96	95	93	92	92	91	91	90
IMP-40	96	95	92	92	91	91	91	90
IMP-77	96	95	92	92	91	91	91	90
IMP-10	96	96	93	92	92	91	91	90
IMP-88	96	95	92	92	91	91	91	90
IMP-55	93	93	91	91	90	90	89	89
IMP-38	99	99	91	91	91	91	90	90
IMP-4	99	99	91	91	92	91	90	90
IMP-59	99	99	91	91	91	91	90	89
IMP-26	100	99	91	91	91	91	90	89
IMP-89	99	100	91	90	91	91	89	89
IMP-5	91	91	100	99	97	97	96	95
IMP-85	91	90	99	100	97	96	95	94
IMP-28	91	91	97	97	100	99	95	93
IMP-94	91	91	97	96	99	100	95	92
IMP-81	90	89	96	95	95	95	100	93
IMP-51	89	89	95	94	93	92	93	100
IMP-43	90	90	95	94	93	92	93	99
IMP-7	90	89	95	95	93	93	94	99
IMP-73	90	89	95	94	93	92	93	99
IMP-15	89	88	93	93	93	92	93	91
IMP-62	88	88	93	93	92	92	92	91
IMP-29	87	87	93	93	91	91	91	88
IMP-82	87	86	93	93	91	91	92	91
IMP-9	86	86	92	92	90	90	91	90
IMP-45	86	86	91	91	90	90	91	90
IMP-53	86	85	91	91	90	89	90	90
IMP-21	85	84	88	88	87	87	89	86
IMP-11	85	84	89	89	88	87	89	87
IMP-68	84	84	88	88	87	87	89	87
IMP-41	85	85	88	88	87	87	89	86
IMP-44	85	84	88	88	87	86	88	86
IMP-22	84	83	87	87	86	86	88	85
IMP-58	84	84	87	87	86	85	87	85
IMP-16	83	83	87	86	85	85	88	85

IMP-74	83	83	86	86	85	85	87	85
IMP-93	83	83	86	86	85	85	87	85
IMP-2	85	84	88	87	87	87	88	87
IMP-19	85	84	88	87	87	87	88	87
IMP-8	85	85	88	88	88	87	89	87
IMP-24	85	84	88	87	87	87	88	87
IMP-96	85	84	88	87	87	87	88	88
IMP-69	85	85	88	88	88	87	89	87
IMP-20	85	85	87	87	87	87	88	87
IMP-23	85	85	88	87	87	87	88	87
IMP-39	83	83	87	87	87	86	87	86
IMP-33	83	83	86	85	85	85	86	85
IMP-17	83	82	85	84	84	84	85	84
IMP-13	83	82	84	84	84	83	85	84
IMP-37	82	82	83	84	83	83	84	83
IMP-84	83	83	84	83	83	83	84	83
IMP-90	81	80	83	83	83	82	86	83
IMP-12	81	80	83	83	83	83	86	84
IMP-63	81	81	83	83	84	83	86	83
IMP-48	81	81	82	82	82	82	82	81
IMP-32	81	81	82	82	82	82	82	81
IMP-14	81	81	82	82	82	82	82	81
IMP-65	80	81	82	81	82	81	82	81
IMP-54	81	81	82	82	82	82	82	81
IMP-91	80	81	82	81	82	81	82	81
IMP-86	81	81	82	82	82	82	82	81
IMP-87	81	81	82	82	82	82	82	81
IMP-83	80	80	81	81	80	80	81	80
IMP-18	80	80	81	81	80	80	81	80
IMP-56	80	80	80	81	80	80	80	80
IMP-49	80	81	80	81	80	80	80	79
IMP-71	80	81	80	81	80	80	80	79
IMP-75	81	81	81	81	80	80	81	80
IMP-46	79	80	80	80	80	79	80	79
IMP-35	81	80	84	84	84	83	83	82
IMP-31	81	80	82	82	82	82	81	81
IMP-92	81	80	82	82	82	82	81	81
IMP-95	80	79	82	82	82	81	83	83
IMP-27	80	79	82	81	81	81	83	82
IMP-64	80	80	82	82	82	81	82	81
IMP-67	79	79	81	81	81	80	82	81

Table S3. (continued)								
Identity (%)	IMP-43	IMP-7	IMP-73	IMP-15	IMP-62	IMP-29	IMP-82	IMP-9
IMP-52	90	90	90	89	89	88	87	86
IMP-42	90	91	90	89	89	88	87	86
IMP-79	90	91	90	89	89	89	87	86
IMP-70	90	91	90	90	89	89	88	87
IMP-66	90	91	90	89	89	88	87	86
IMP-60	90	91	90	89	89	88	87	86
IMP-30	90	91	90	89	89	88	87	86
IMP-34	90	91	90	89	89	88	87	86
IMP-1	91	91	91	90	89	89	88	87
IMP-61	90	91	90	89	89	88	87	86
IMP-80	91	90	90	89	89	88	87	86
IMP-78	91	90	90	89	89	88	87	86
IMP-6	90	91	90	89	90	88	87	86
IMP-3	90	90	90	89	89	88	87	86
IMP-25	90	90	90	89	89	88	87	86
IMP-76	91	91	91	89	89	88	87	87
IMP-40	91	90	90	89	89	88	87	86
IMP-77	91	90	90	89	89	88	87	86
IMP-10	91	91	91	89	89	88	87	87
IMP-88	91	90	90	89	89	88	87	86
IMP-55	89	89	89	88	87	87	86	85
IMP-38	89	90	89	89	89	87	87	86
IMP-4	90	90	90	89	89	87	87	86
IMP-59	89	90	89	89	88	87	87	86
IMP-26	90	90	90	89	88	87	87	86
IMP-89	90	89	89	88	88	87	86	86
IMP-5	95	95	95	93	93	93	93	92
IMP-85	94	95	94	93	93	93	93	92
IMP-28	93	93	93	93	92	91	91	90
IMP-94	92	93	92	92	92	91	91	90
IMP-81	93	94	93	93	92	91	92	91
IMP-51	99	99	99	91	91	88	91	90
IMP-43	100	99	99	91	91	88	91	90
IMP-7	99	100	99	91	91	88	91	90
IMP-73	99	99	100	91	91	88	91	90
IMP-15	91	91	91	100	99	92	90	90
IMP-62	91	91	91	99	100	91	90	89
IMP-29	88	88	88	92	91	100	90	89
IMP-82	91	91	91	90	90	90	100	98
IMP-9	90	90	90	90	89	89	98	100
IMP-45	90	90	90	89	90	89	98	99
IMP-53	90	90	90	89	89	88	98	99
IMP-21	87	87	87	87	87	91	88	87
IMP-11	87	87	87	88	87	91	88	87
IMP-68	86	87	86	87	88	91	88	87
IMP-41	87	87	87	87	87	91	88	87
IMP-44	87	86	86	87	86	90	87	87
IMP-22	85	85	85	87	87	92	86	85
IMP-58	85	85	85	87	87	91	86	85
IMP-16	85	85	85	86	86	91	85	84

IMP-74	85	85	85	86	85	91	85	84
IMP-93	85	85	85	86	85	91	85	84
IMP-2	87	87	87	88	88	86	87	87
IMP-19	87	87	87	88	88	86	87	87
IMP-8	87	88	87	89	88	86	87	86
IMP-24	87	87	87	88	88	86	86	86
IMP-96	87	87	87	88	89	86	86	86
IMP-69	87	87	87	89	88	86	88	87
IMP-20	87	87	87	88	87	85	86	86
IMP-23	88	87	87	88	88	86	86	86
IMP-39	86	86	86	89	89	85	85	85
IMP-33	85	85	85	87	87	84	84	84
IMP-17	84	85	84	86	85	83	83	83
IMP-13	84	84	84	85	85	83	82	83
IMP-37	83	83	83	85	84	82	82	83
IMP-84	84	84	84	85	85	82	82	83
IMP-90	83	83	83	86	85	85	83	83
IMP-12	83	83	83	86	86	85	83	83
IMP-63	83	84	83	86	86	85	83	83
IMP-48	81	82	81	82	82	81	83	82
IMP-32	81	82	81	82	82	81	83	82
IMP-14	81	82	81	82	82	81	83	82
IMP-65	81	81	81	82	81	80	82	82
IMP-54	81	82	81	82	82	81	83	82
IMP-91	81	81	81	82	81	80	82	82
IMP-86	81	82	81	82	82	81	83	82
IMP-87	81	82	81	82	82	81	83	82
IMP-83	80	80	80	82	81	80	80	80
IMP-18	80	80	80	82	81	80	80	80
IMP-56	79	80	79	81	82	79	80	80
IMP-49	80	80	80	81	81	79	80	80
IMP-71	80	80	80	81	81	79	80	80
IMP-75	80	80	80	81	80	80	80	81
IMP-46	79	79	79	79	79	78	79	78
IMP-35	83	83	83	84	84	84	82	82
IMP-31	81	81	81	82	82	82	82	82
IMP-92	81	81	81	82	82	82	82	82
IMP-95	82	82	82	83	84	81	81	80
IMP-27	81	81	81	83	83	80	81	80
IMP-64	80	81	80	83	83	81	80	80
IMP-67	80	81	80	83	83	80	81	80

Table S3. (continued)								
**Identity (%)**	**IMP-45**	**IMP-53**	**IMP-21**	**IMP-11**	**IMP-68**	**IMP-41**	**IMP-44**	**IMP-22**
IMP-52	86	85	86	86	86	86	85	85
IMP-42	86	86	86	87	86	86	86	85
IMP-79	86	86	87	87	87	87	86	85
IMP-70	86	86	87	87	87	87	86	85
IMP-66	86	86	86	87	86	86	86	85
IMP-60	86	86	87	88	87	87	87	85
IMP-30	86	86	86	87	86	86	86	85
IMP-34	86	86	86	87	86	86	86	85
IMP-1	86	86	87	87	87	87	86	85
IMP-61	86	86	86	87	86	86	86	85
IMP-80	86	86	86	86	86	87	86	85
IMP-78	87	86	86	86	87	87	86	85
IMP-6	87	86	86	87	87	86	86	85
IMP-3	86	86	86	86	87	86	85	85
IMP-25	86	86	86	87	87	86	86	85
IMP-76	86	86	87	87	86	87	86	85
IMP-40	86	86	86	86	86	87	87	85
IMP-77	86	86	87	87	86	87	87	85
IMP-10	86	86	87	87	86	87	87	85
IMP-88	86	86	86	86	86	87	86	85
IMP-55	84	84	85	86	85	85	85	84
IMP-38	86	86	84	85	85	84	84	84
IMP-4	86	86	85	85	85	85	84	84
IMP-59	86	85	84	85	84	84	84	84
IMP-26	86	86	85	85	84	85	85	84
IMP-89	86	85	84	84	84	85	84	83
IMP-5	91	91	88	89	88	88	88	87
IMP-85	91	91	88	89	88	88	88	87
IMP-28	90	90	87	88	87	87	87	86
IMP-94	90	89	87	87	87	87	86	86
IMP-81	91	90	89	89	89	89	88	88
IMP-51	90	90	86	87	87	86	86	85
IMP-43	90	90	87	87	86	87	87	85
IMP-7	90	90	87	87	87	87	86	85
IMP-73	90	90	87	87	86	87	86	85
IMP-15	89	89	87	88	87	87	87	87
IMP-62	90	89	87	87	88	87	86	87
IMP-29	89	88	91	91	91	91	90	92
IMP-82	98	98	88	88	88	88	87	86
IMP-9	99	99	87	87	87	87	87	85
IMP-45	100	99	87	87	87	87	87	85
IMP-53	99	100	87	87	87	87	86	84
IMP-21	87	87	100	99	99	99	99	93
IMP-11	87	87	99	100	99	99	99	94
IMP-68	87	87	99	99	100	99	99	93
IMP-41	87	87	99	99	99	100	99	93
IMP-44	87	86	99	99	99	99	100	93
IMP-22	85	84	93	94	93	93	93	100
IMP-58	85	84	93	93	93	94	93	99
IMP-16	84	83	93	93	93	93	92	94

**IMP-74**	**84**	**83**	**93**	**93**	**92**	**93**	**93**	**94**
**IMP-93**	**84**	**83**	**93**	**93**	**92**	**93**	**93**	**94**
**IMP-2**	**86**	**85**	**90**	**91**	**90**	**90**	**90**	**89**
**IMP-19**	**86**	**85**	**90**	**91**	**90**	**90**	**90**	**89**
**IMP-8**	**86**	**85**	**91**	**91**	**91**	**91**	**90**	**90**
**IMP-24**	**85**	**85**	**90**	**91**	**90**	**90**	**90**	**89**
**IMP-96**	**86**	**86**	**90**	**91**	**91**	**90**	**90**	**89**
**IMP-69**	**86**	**85**	**91**	**91**	**91**	**91**	**90**	**90**
**IMP-20**	**86**	**85**	**90**	**90**	**90**	**91**	**90**	**89**
**IMP-23**	**86**	**85**	**91**	**91**	**90**	**91**	**91**	**89**
**IMP-39**	**84**	**84**	**89**	**90**	**89**	**89**	**89**	**88**
**IMP-33**	**84**	**83**	**86**	**87**	**86**	**86**	**86**	**87**
**IMP-17**	**83**	**82**	**85**	**86**	**85**	**85**	**85**	**86**
**IMP-13**	**82**	**82**	**85**	**86**	**85**	**85**	**85**	**86**
**IMP-37**	**82**	**82**	**85**	**86**	**85**	**85**	**85**	**85**
**IMP-84**	**82**	**82**	**85**	**85**	**85**	**86**	**85**	**85**
**IMP-90**	**82**	**82**	**86**	**87**	**86**	**86**	**87**	**87**
**IMP-12**	**83**	**83**	**86**	**87**	**87**	**86**	**86**	**87**
**IMP-63**	**83**	**82**	**87**	**87**	**87**	**87**	**86**	**87**
**IMP-48**	**82**	**81**	**84**	**85**	**84**	**84**	**84**	**84**
**IMP-32**	**82**	**81**	**84**	**85**	**84**	**84**	**84**	**84**
**IMP-14**	**82**	**81**	**84**	**85**	**84**	**84**	**84**	**84**
**IMP-65**	**81**	**81**	**84**	**84**	**84**	**84**	**84**	**83**
**IMP-54**	**82**	**81**	**84**	**85**	**84**	**84**	**84**	**84**
**IMP-91**	**81**	**81**	**84**	**84**	**84**	**84**	**84**	**83**
**IMP-86**	**82**	**81**	**84**	**85**	**84**	**84**	**84**	**84**
**IMP-87**	**82**	**81**	**84**	**85**	**84**	**84**	**84**	**84**
**IMP-83**	**80**	**80**	**83**	**83**	**83**	**83**	**83**	**82**
**IMP-18**	**80**	**80**	**83**	**83**	**83**	**83**	**83**	**82**
**IMP-56**	**80**	**80**	**83**	**83**	**83**	**83**	**83**	**81**
**IMP-49**	**80**	**80**	**83**	**83**	**83**	**83**	**83**	**81**
**IMP-71**	**80**	**80**	**83**	**83**	**83**	**83**	**83**	**81**
**IMP-75**	**80**	**80**	**83**	**83**	**83**	**84**	**84**	**82**
**IMP-46**	**78**	**78**	**81**	**82**	**81**	**81**	**81**	**81**
**IMP-35**	**82**	**81**	**85**	**85**	**85**	**85**	**85**	**86**
**IMP-31**	**82**	**81**	**83**	**83**	**83**	**83**	**83**	**84**
**IMP-92**	**82**	**81**	**83**	**83**	**83**	**83**	**83**	**84**
**IMP-95**	**81**	**80**	**84**	**84**	**85**	**84**	**83**	**83**
**IMP-27**	**80**	**80**	**83**	**83**	**83**	**83**	**82**	**83**
**IMP-64**	**80**	**80**	**82**	**83**	**83**	**82**	**82**	**83**
**IMP-67**	**81**	**80**	**82**	**83**	**83**	**82**	**82**	**83**

Table S3. (continued)								
**Identity (%)**	**IMP-58**	**IMP-16**	**IMP-74**	**IMP-93**	**IMP-2**	**IMP-19**	**IMP-8**	**IMP-24**
**IMP-52**	**84**	**84**	**84**	**84**	**85**	**85**	**85**	**85**
**IMP-42**	**85**	**85**	**84**	**84**	**85**	**85**	**85**	**85**
**IMP-79**	**85**	**85**	**85**	**85**	**85**	**85**	**85**	**85**
**IMP-70**	**85**	**85**	**85**	**85**	**85**	**85**	**86**	**85**
**IMP-66**	**85**	**85**	**84**	**84**	**85**	**85**	**85**	**85**
**IMP-60**	**85**	**85**	**84**	**84**	**85**	**85**	**85**	**85**
**IMP-30**	**85**	**85**	**84**	**84**	**85**	**85**	**85**	**85**
**IMP-34**	**85**	**85**	**84**	**84**	**85**	**85**	**85**	**85**
**IMP-1**	**85**	**85**	**85**	**85**	**85**	**85**	**86**	**85**
**IMP-61**	**85**	**85**	**84**	**84**	**85**	**85**	**85**	**85**
**IMP-80**	**85**	**84**	**85**	**85**	**85**	**85**	**85**	**85**
**IMP-78**	**85**	**84**	**85**	**85**	**85**	**85**	**85**	**85**
**IMP-6**	**85**	**85**	**84**	**84**	**85**	**85**	**85**	**85**
**IMP-3**	**84**	**84**	**84**	**84**	**85**	**85**	**85**	**85**
**IMP-25**	**85**	**85**	**84**	**84**	**85**	**85**	**85**	**85**
**IMP-76**	**85**	**85**	**85**	**85**	**85**	**85**	**85**	**85**
**IMP-40**	**85**	**84**	**85**	**85**	**85**	**85**	**85**	**85**
**IMP-77**	**85**	**85**	**85**	**85**	**85**	**85**	**85**	**85**
**IMP-10**	**85**	**85**	**85**	**85**	**85**	**85**	**85**	**85**
**IMP-88**	**85**	**85**	**85**	**85**	**85**	**85**	**85**	**85**
**IMP-55**	**84**	**84**	**83**	**83**	**85**	**85**	**85**	**85**
**IMP-38**	**83**	**83**	**83**	**83**	**85**	**85**	**85**	**85**
**IMP-4**	**84**	**83**	**83**	**83**	**85**	**85**	**85**	**85**
**IMP-59**	**83**	**83**	**83**	**83**	**85**	**85**	**85**	**85**
**IMP-26**	**84**	**83**	**83**	**83**	**85**	**85**	**85**	**85**
**IMP-89**	**84**	**83**	**83**	**83**	**84**	**84**	**85**	**84**
**IMP-5**	**87**	**87**	**86**	**86**	**88**	**88**	**88**	**88**
**IMP-85**	**87**	**86**	**86**	**86**	**87**	**87**	**88**	**87**
**IMP-28**	**86**	**85**	**85**	**85**	**87**	**87**	**88**	**87**
**IMP-94**	**85**	**85**	**85**	**85**	**87**	**87**	**87**	**87**
**IMP-81**	**87**	**88**	**87**	**87**	**88**	**88**	**89**	**88**
**IMP-51**	**85**	**85**	**85**	**85**	**87**	**87**	**87**	**87**
**IMP-43**	**85**	**85**	**85**	**85**	**87**	**87**	**87**	**87**
**IMP-7**	**85**	**85**	**85**	**85**	**87**	**87**	**88**	**87**
**IMP-73**	**85**	**85**	**85**	**85**	**87**	**87**	**87**	**87**
**IMP-15**	**87**	**86**	**86**	**86**	**88**	**88**	**89**	**88**
**IMP-62**	**87**	**86**	**85**	**85**	**88**	**88**	**88**	**88**
**IMP-29**	**91**	**91**	**91**	**91**	**86**	**86**	**86**	**86**
**IMP-82**	**86**	**85**	**85**	**85**	**87**	**87**	**87**	**86**
**IMP-9**	**85**	**84**	**84**	**84**	**87**	**87**	**86**	**86**
**IMP-45**	**85**	**84**	**84**	**84**	**86**	**86**	**86**	**85**
**IMP-53**	**84**	**83**	**83**	**83**	**85**	**85**	**85**	**85**
**IMP-21**	**93**	**93**	**93**	**93**	**90**	**90**	**91**	**90**
**IMP-11**	**93**	**93**	**93**	**93**	**91**	**91**	**91**	**91**
**IMP-68**	**93**	**93**	**92**	**92**	**90**	**90**	**91**	**90**
**IMP-41**	**94**	**93**	**93**	**93**	**90**	**90**	**91**	**90**
**IMP-44**	**93**	**92**	**93**	**93**	**90**	**90**	**90**	**90**
**IMP-22**	**99**	**94**	**94**	**94**	**89**	**89**	**90**	**89**
**IMP-58**	**100**	**94**	**94**	**94**	**89**	**89**	**89**	**89**
**IMP-16**	**94**	**100**	**99**	**99**	**88**	**88**	**89**	**88**

IMP-74	94	99	100	99	88	88	88	88
IMP-93	94	99	99	100	88	88	88	88
IMP-2	89	88	88	88	100	99	99	99
IMP-19	89	88	88	88	99	100	99	99
IMP-8	89	89	88	88	99	99	100	99
IMP-24	89	88	88	88	99	99	99	100
IMP-96	89	88	88	88	99	99	99	99
IMP-69	89	89	88	88	99	99	99	99
IMP-20	89	88	88	88	99	99	99	99
IMP-23	90	88	89	89	99	99	99	99
IMP-39	88	87	87	87	96	96	97	96
IMP-33	87	87	87	86	93	93	93	93
IMP-17	85	85	85	85	92	93	93	93
IMP-13	85	85	85	85	92	93	93	93
IMP-37	85	85	84	84	91	92	92	92
IMP-84	86	85	85	85	92	92	93	92
IMP-90	86	87	86	86	87	87	87	87
IMP-12	86	87	86	86	87	87	87	87
IMP-63	87	87	87	86	87	87	87	87
IMP-48	83	84	83	83	88	88	88	88
IMP-32	83	84	83	83	88	88	88	88
IMP-14	83	84	83	83	88	88	88	88
IMP-65	83	84	83	83	87	87	88	87
IMP-54	83	84	83	83	88	88	88	88
IMP-91	83	83	83	83	87	87	88	87
IMP-86	83	84	83	83	88	88	88	88
IMP-87	83	84	83	83	88	88	88	88
IMP-83	81	82	82	81	88	88	88	88
IMP-18	81	82	82	81	88	88	88	88
IMP-56	81	82	81	81	87	87	88	87
IMP-49	82	82	82	82	87	87	88	87
IMP-71	82	81	82	82	87	87	88	87
IMP-75	82	82	82	83	87	87	87	87
IMP-46	80	81	81	80	86	86	86	85
IMP-35	86	85	85	85	86	86	87	86
IMP-31	84	83	83	83	84	84	84	84
IMP-92	84	83	83	83	84	84	84	84
IMP-95	83	84	83	83	89	90	90	90
IMP-27	83	83	83	83	87	87	87	87
IMP-64	82	83	83	82	87	87	87	87
IMP-67	82	83	83	82	87	87	87	87

Table S3. (continued)								
Identity (%)	IMP-96	IMP-69	IMP-20	IMP-23	IMP-39	IMP-33	IMP-17	IMP-13
IMP-52	85	85	84	85	83	83	82	82
IMP-42	85	85	85	85	84	83	82	82
IMP-79	85	85	85	85	84	83	82	82
IMP-70	85	86	85	85	84	83	83	83
IMP-66	85	85	85	85	84	83	82	82
IMP-60	85	85	85	85	84	83	82	82
IMP-30	85	85	85	85	84	83	82	82
IMP-34	85	85	85	85	84	83	82	82
IMP-1	85	86	85	85	84	83	83	83
IMP-61	85	85	85	85	84	83	82	82
IMP-80	85	85	85	85	83	83	82	82
IMP-78	85	85	85	85	83	83	82	82
IMP-6	86	85	85	85	84	83	82	82
IMP-3	85	85	84	85	83	83	82	82
IMP-25	85	85	84	85	83	83	82	82
IMP-76	85	85	85	85	84	83	82	82
IMP-40	85	85	85	85	83	83	82	82
IMP-77	85	85	85	86	84	83	82	82
IMP-10	85	85	85	86	84	83	82	82
IMP-88	85	85	85	85	83	83	82	82
IMP-55	85	85	84	85	83	82	81	81
IMP-38	85	85	84	85	83	83	83	83
IMP-4	85	85	85	85	84	83	83	83
IMP-59	85	85	84	85	83	83	83	83
IMP-26	85	85	85	85	83	83	83	83
IMP-89	84	85	85	85	83	83	82	82
IMP-5	88	88	87	88	87	86	85	84
IMP-85	87	88	87	87	87	85	84	84
IMP-28	87	88	87	87	87	85	84	84
IMP-94	87	87	87	87	86	85	84	83
IMP-81	88	89	88	88	87	86	85	85
IMP-51	88	87	87	87	86	85	84	84
IMP-43	87	87	87	88	86	85	84	84
IMP-7	87	87	87	87	86	85	85	84
IMP-73	87	87	87	87	86	85	84	84
IMP-15	88	89	88	88	89	87	86	85
IMP-62	89	88	87	88	89	87	85	85
IMP-29	86	86	85	86	85	84	83	83
IMP-82	86	88	86	86	85	84	83	82
IMP-9	86	87	86	86	85	84	83	83
IMP-45	86	86	86	86	84	84	83	82
IMP-53	86	85	85	85	84	83	82	82
IMP-21	90	91	90	91	89	86	85	85
IMP-11	91	91	90	91	90	87	86	86
IMP-68	91	91	90	90	89	86	85	85
IMP-41	90	91	91	91	89	86	85	85
IMP-44	90	90	90	91	89	86	85	85
IMP-22	89	90	89	89	88	87	86	86
IMP-58	89	89	89	90	88	87	85	85
IMP-16	88	89	88	88	87	87	85	85

**IMP-74**	**88**	**88**	**88**	**89**	**87**	**87**	**85**	**85**
**IMP-93**	**88**	**88**	**88**	**89**	**87**	**86**	**85**	**85**
**IMP-2**	**99**	**99**	**99**	**99**	**96**	**93**	**92**	**92**
**IMP-19**	**99**	**99**	**99**	**99**	**96**	**93**	**93**	**93**
**IMP-8**	**99**	**99**	**99**	**99**	**97**	**93**	**93**	**93**
**IMP-24**	**99**	**99**	**99**	**99**	**96**	**93**	**93**	**93**
**IMP-96**	**100**	**99**	**99**	**99**	**96**	**93**	**93**	**93**
**IMP-69**	**99**	**100**	**99**	**99**	**96**	**93**	**93**	**93**
**IMP-20**	**99**	**99**	**100**	**99**	**96**	**93**	**92**	**92**
**IMP-23**	**99**	**99**	**99**	**100**	**96**	**93**	**93**	**93**
**IMP-39**	**96**	**96**	**96**	**96**	**100**	**94**	**94**	**94**
**IMP-33**	**93**	**93**	**93**	**93**	**94**	**100**	**97**	**98**
**IMP-17**	**93**	**93**	**92**	**93**	**94**	**97**	**100**	**99**
**IMP-13**	**93**	**93**	**92**	**93**	**94**	**98**	**99**	**100**
**IMP-37**	**92**	**92**	**91**	**92**	**93**	**98**	**99**	**99**
**IMP-84**	**92**	**92**	**93**	**93**	**93**	**98**	**99**	**99**
**IMP-90**	**87**	**87**	**86**	**87**	**86**	**85**	**84**	**84**
**IMP-12**	**87**	**87**	**86**	**87**	**86**	**85**	**84**	**84**
**IMP-63**	**87**	**87**	**87**	**87**	**87**	**85**	**85**	**85**
**IMP-48**	**88**	**88**	**87**	**88**	**86**	**85**	**84**	**84**
**IMP-32**	**88**	**88**	**87**	**88**	**86**	**85**	**84**	**84**
**IMP-14**	**88**	**88**	**87**	**88**	**86**	**85**	**84**	**84**
**IMP-65**	**87**	**88**	**87**	**87**	**85**	**85**	**83**	**83**
**IMP-54**	**88**	**88**	**87**	**88**	**86**	**85**	**84**	**84**
**IMP-91**	**87**	**88**	**87**	**87**	**85**	**85**	**83**	**83**
**IMP-86**	**88**	**88**	**87**	**88**	**86**	**85**	**84**	**84**
**IMP-87**	**88**	**88**	**87**	**88**	**86**	**85**	**84**	**84**
**IMP-83**	**88**	**88**	**87**	**88**	**85**	**84**	**83**	**83**
**IMP-18**	**88**	**88**	**87**	**88**	**85**	**84**	**83**	**83**
**IMP-56**	**88**	**88**	**87**	**87**	**85**	**83**	**82**	**82**
**IMP-49**	**87**	**88**	**88**	**88**	**85**	**83**	**82**	**82**
**IMP-71**	**87**	**88**	**88**	**88**	**85**	**83**	**82**	**82**
**IMP-75**	**87**	**87**	**87**	**88**	**85**	**83**	**81**	**81**
**IMP-46**	**85**	**86**	**85**	**85**	**83**	**83**	**81**	**81**
**IMP-35**	**86**	**87**	**86**	**87**	**85**	**84**	**83**	**83**
**IMP-31**	**84**	**84**	**84**	**84**	**83**	**83**	**82**	**82**
**IMP-92**	**84**	**84**	**84**	**84**	**83**	**83**	**82**	**82**
**IMP-95**	**91**	**90**	**89**	**90**	**89**	**89**	**89**	**89**
**IMP-27**	**88**	**88**	**87**	**87**	**87**	**86**	**86**	**85**
**IMP-64**	**87**	**87**	**86**	**87**	**86**	**86**	**85**	**85**
**IMP-67**	**87**	**87**	**86**	**87**	**86**	**86**	**85**	**85**

Table S3. (continued)								
**Identity (%)**	**IMP-37**	**IMP-84**	**IMP-90**	**IMP-12**	**IMP-63**	**IMP-48**	**IMP-32**	**IMP-14**
**IMP-52**	81	81	82	82	82	80	80	80
**IMP-42**	81	82	82	82	83	81	81	81
**IMP-79**	81	82	82	82	83	81	81	81
**IMP-70**	82	82	83	83	83	81	81	81
**IMP-66**	81	82	82	82	83	81	81	81
**IMP-60**	81	82	82	82	83	81	81	81
**IMP-30**	81	82	82	82	83	81	81	81
**IMP-34**	81	82	82	82	83	81	81	81
**IMP-1**	82	82	83	83	83	81	81	81
**IMP-61**	81	82	82	82	83	81	81	81
**IMP-80**	81	82	82	82	82	80	80	80
**IMP-78**	81	82	82	83	82	80	80	80
**IMP-6**	81	82	82	83	83	81	81	81
**IMP-3**	81	81	82	83	82	80	80	80
**IMP-25**	81	81	82	83	83	80	80	80
**IMP-76**	81	82	82	82	83	81	81	81
**IMP-40**	81	82	83	82	82	81	81	81
**IMP-77**	81	83	82	82	83	81	81	81
**IMP-10**	81	83	82	82	83	81	81	81
**IMP-88**	81	83	82	82	83	80	80	80
**IMP-55**	80	81	81	81	82	80	80	80
**IMP-38**	82	82	81	82	81	81	81	81
**IMP-4**	83	83	81	81	82	81	81	81
**IMP-59**	82	82	81	81	81	82	82	82
**IMP-26**	82	83	81	81	81	81	81	81
**IMP-89**	82	83	80	80	81	81	81	81
**IMP-5**	83	84	83	83	83	82	82	82
**IMP-85**	84	83	83	83	83	82	82	82
**IMP-28**	83	83	83	83	84	82	82	82
**IMP-94**	83	83	82	83	83	82	82	82
**IMP-81**	84	84	86	86	86	82	82	82
**IMP-51**	83	83	83	84	83	81	81	81
**IMP-43**	83	84	83	83	83	81	81	81
**IMP-7**	83	84	83	83	84	82	82	82
**IMP-73**	83	84	83	83	83	81	81	81
**IMP-15**	85	85	86	86	86	82	82	82
**IMP-62**	84	85	85	86	86	82	82	82
**IMP-29**	82	82	85	85	85	81	81	81
**IMP-82**	82	82	83	83	83	83	83	83
**IMP-9**	83	83	83	83	83	82	82	82
**IMP-45**	82	82	82	83	83	82	82	82
**IMP-53**	82	82	82	83	82	81	81	81
**IMP-21**	85	85	86	86	87	84	84	84
**IMP-11**	86	85	87	87	87	85	85	85
**IMP-68**	85	85	86	87	87	84	84	84
**IMP-41**	85	86	86	86	87	84	84	84
**IMP-44**	85	85	87	86	86	84	84	84
**IMP-22**	85	85	87	87	87	84	84	84
**IMP-58**	85	86	86	86	87	83	83	83
**IMP-16**	85	85	87	87	87	84	84	84

IMP-74	84	85	86	86	87	83	83	83
IMP-93	84	85	86	86	86	83	83	83
IMP-2	91	92	87	87	87	88	88	88
IMP-19	92	92	87	87	87	88	88	88
IMP-8	92	93	87	87	87	88	88	88
IMP-24	92	92	87	87	87	88	88	88
IMP-96	92	92	87	87	87	88	88	88
IMP-69	92	92	87	87	87	88	88	88
IMP-20	91	93	86	86	87	87	87	87
IMP-23	92	93	87	87	87	88	88	88
IMP-39	93	93	86	86	87	86	86	86
IMP-33	98	98	85	85	85	85	85	85
IMP-17	99	99	84	84	85	84	84	84
IMP-13	99	99	84	84	85	84	84	84
IMP-37	100	99	85	85	85	84	84	84
IMP-84	99	100	84	84	84	83	83	83
IMP-90	85	84	100	98	98	82	82	82
IMP-12	85	84	98	100	99	82	82	82
IMP-63	85	84	98	99	100	82	82	82
IMP-48	84	83	82	82	82	100	99	99
IMP-32	84	83	82	82	82	99	100	99
IMP-14	84	83	82	82	82	99	99	100
IMP-65	83	83	82	81	82	99	99	99
IMP-54	84	83	82	82	82	99	99	99
IMP-91	83	83	82	81	82	99	99	99
IMP-86	84	83	82	82	82	99	98	99
IMP-87	84	83	82	82	82	99	99	99
IMP-83	82	82	84	83	84	91	91	91
IMP-18	82	82	84	83	84	91	92	92
IMP-56	82	82	83	84	83	91	91	91
IMP-49	82	83	83	83	83	91	91	91
IMP-71	81	82	83	83	83	91	91	91
IMP-75	81	82	83	82	83	90	91	91
IMP-46	81	80	82	81	82	91	91	91
IMP-35	84	83	83	83	84	81	81	81
IMP-31	82	82	82	82	83	80	80	80
IMP-92	82	82	82	82	83	80	80	80
IMP-95	88	88	83	84	83	84	84	84
IMP-27	84	85	83	83	83	84	84	84
IMP-64	84	84	82	83	83	84	84	84
IMP-67	84	84	82	83	83	84	84	84

Table S3. (continued)								
Identity (%)	IMP-65	IMP-54	IMP-91	IMP-86	IMP-87	IMP-83	IMP-18	IMP-56
IMP-52	80	80	80	80	80	80	80	79
IMP-42	80	81	80	81	81	80	80	80
IMP-79	80	81	80	81	81	80	80	80
IMP-70	81	81	81	81	81	80	80	80
IMP-66	80	81	80	81	81	80	80	80
IMP-60	80	81	80	81	81	80	80	80
IMP-30	80	81	80	81	81	80	80	80
IMP-34	80	81	80	81	81	80	80	80
IMP-1	81	81	81	81	81	80	80	80
IMP-61	80	81	80	81	81	80	80	80
IMP-80	80	80	80	80	80	80	80	80
IMP-78	80	80	80	80	80	80	80	80
IMP-6	80	81	80	81	81	80	80	80
IMP-3	80	80	80	80	80	80	80	80
IMP-25	80	80	80	80	80	80	80	80
IMP-76	80	81	80	81	81	80	80	80
IMP-40	80	81	80	81	81	80	80	80
IMP-77	80	81	80	81	81	80	80	80
IMP-10	80	81	80	81	81	80	80	80
IMP-88	80	80	80	80	80	80	80	79
IMP-55	80	80	80	80	80	80	80	79
IMP-38	80	81	80	81	81	80	80	80
IMP-4	81	81	81	81	81	80	80	80
IMP-59	81	82	81	82	82	80	80	80
IMP-26	80	81	80	81	81	80	80	80
IMP-89	81	81	81	81	81	80	80	80
IMP-5	82	82	82	82	82	81	81	80
IMP-85	81	82	81	82	82	81	81	81
IMP-28	82	82	82	82	82	80	80	80
IMP-94	81	82	81	82	82	80	80	80
IMP-81	82	82	82	82	82	81	81	80
IMP-51	81	81	81	81	81	80	80	80
IMP-43	81	81	81	81	81	80	80	79
IMP-7	81	82	81	82	82	80	80	80
IMP-73	81	81	81	81	81	80	80	79
IMP-15	82	82	82	82	82	82	82	81
IMP-62	81	82	81	82	82	81	81	82
IMP-29	80	81	80	81	81	80	80	79
IMP-82	82	83	82	83	83	80	80	80
IMP-9	82	82	82	82	82	80	80	80
IMP-45	81	82	81	82	82	80	80	80
IMP-53	81	81	81	81	81	80	80	80
IMP-21	84	84	84	84	84	83	83	83
IMP-11	84	85	84	85	85	83	83	83
IMP-68	84	84	84	84	84	83	83	83
IMP-41	84	84	84	84	84	83	83	83
IMP-44	84	84	84	84	84	83	83	83
IMP-22	83	84	83	84	84	82	82	81
IMP-58	83	83	83	83	83	81	81	81
IMP-16	84	84	83	84	84	82	82	82

IMP-74	83	83	83	83	83	82	82	81
IMP-93	83	83	83	83	83	81	81	81
IMP-2	87	88	87	88	88	88	88	87
IMP-19	87	88	87	88	88	88	88	87
IMP-8	88	88	88	88	88	88	88	88
IMP-24	87	88	87	88	88	88	88	87
IMP-96	87	88	87	88	88	88	88	88
IMP-69	88	88	88	88	88	88	88	88
IMP-20	87	87	87	87	87	87	87	87
IMP-23	87	88	87	88	88	88	88	87
IMP-39	85	86	85	86	86	85	85	85
IMP-33	85	85	85	85	85	84	84	83
IMP-17	83	84	83	84	84	83	83	82
IMP-13	83	84	83	84	84	83	83	82
IMP-37	83	84	83	84	84	82	82	82
IMP-84	83	83	83	83	83	82	82	82
IMP-90	82	82	82	82	82	84	84	83
IMP-12	81	82	81	82	82	83	83	84
IMP-63	82	82	82	82	82	84	84	83
IMP-48	99	99	99	99	99	91	91	91
IMP-32	99	99	99	98	99	91	92	91
IMP-14	99	99	99	99	99	91	92	91
IMP-65	100	99	99	98	99	91	91	91
IMP-54	99	100	99	99	99	92	91	91
IMP-91	99	99	100	99	99	91	91	91
IMP-86	98	99	99	100	99	92	91	91
IMP-87	99	99	99	99	100	91	92	91
IMP-83	91	92	91	92	91	100	99	99
IMP-18	91	91	91	91	92	99	100	99
IMP-56	91	91	91	91	91	99	99	100
IMP-49	91	91	91	91	91	99	99	99
IMP-71	91	91	91	91	91	99	99	99
IMP-75	90	90	90	90	91	98	99	98
IMP-46	91	91	91	90	91	89	90	89
IMP-35	82	81	81	82	82	80	80	80
IMP-31	81	80	80	81	81	79	79	79
IMP-92	81	80	80	81	81	79	79	79
IMP-95	83	84	83	84	84	83	83	83
IMP-27	83	84	83	84	84	82	82	82
IMP-64	83	84	83	84	84	82	82	82
IMP-67	83	84	83	84	84	81	81	82

Table S3. (continued)								
Identity (%)	IMP-49	IMP-71	IMP-75	IMP-46	IMP-35	IMP-31	IMP-92	IMP-95
IMP-52	79	79	80	79	82	80	80	79
IMP-42	80	80	80	80	82	80	80	79
IMP-79	80	80	80	80	82	80	80	79
IMP-70	80	80	80	80	82	80	80	80
IMP-66	80	80	80	80	82	80	80	79
IMP-60	80	80	80	80	82	80	80	79
IMP-30	80	80	80	80	82	80	80	79
IMP-34	80	80	80	80	82	80	80	79
IMP-1	80	80	80	80	82	80	80	80
IMP-61	80	80	80	80	82	80	80	79
IMP-80	80	80	80	79	82	80	80	79
IMP-78	80	80	80	79	82	80	80	80
IMP-6	80	80	80	80	82	80	80	80
IMP-3	79	79	80	79	81	80	80	80
IMP-25	79	79	80	79	81	80	80	80
IMP-76	80	80	80	80	82	80	80	79
IMP-40	80	80	81	80	82	80	80	79
IMP-77	80	80	81	80	82	80	80	79
IMP-10	80	80	81	80	82	80	80	79
IMP-88	80	80	80	80	82	80	80	79
IMP-55	79	79	80	80	80	79	79	78
IMP-38	80	80	80	79	80	80	80	80
IMP-4	80	80	80	80	81	81	81	80
IMP-59	80	80	80	80	81	81	81	80
IMP-26	80	80	81	79	81	81	81	80
IMP-89	81	81	81	80	80	80	80	79
IMP-5	80	80	81	80	84	82	82	82
IMP-85	81	81	81	80	84	82	82	82
IMP-28	80	80	80	80	84	82	82	82
IMP-94	80	80	80	79	83	82	82	81
IMP-81	80	80	81	80	83	81	81	83
IMP-51	79	79	80	79	82	81	81	83
IMP-43	80	80	80	79	83	81	81	82
IMP-7	80	80	80	79	83	81	81	82
IMP-73	80	80	80	79	83	81	81	82
IMP-15	81	81	81	79	84	82	82	83
IMP-62	81	81	80	79	84	82	82	84
IMP-29	79	79	80	78	84	82	82	81
IMP-82	80	80	80	79	82	82	82	81
IMP-9	80	80	81	78	82	82	82	80
IMP-45	80	80	80	78	82	82	82	81
IMP-53	80	80	80	78	81	81	81	80
IMP-21	83	83	83	81	85	83	83	84
IMP-11	83	83	83	82	85	83	83	84
IMP-68	83	83	83	81	85	83	83	85
IMP-41	83	83	84	81	85	83	83	84
IMP-44	83	83	84	81	85	83	83	83
IMP-22	81	81	82	81	86	84	84	83
IMP-58	82	82	82	80	86	84	84	83
IMP-16	82	81	82	81	85	83	83	84

IMP-74	82	82	82	81	85	83	83	83
IMP-93	82	82	83	80	85	83	83	83
IMP-2	87	87	87	86	86	84	84	89
IMP-19	87	87	87	86	86	84	84	90
IMP-8	88	88	87	86	87	84	84	90
IMP-24	87	87	87	85	86	84	84	90
IMP-96	87	87	87	85	86	84	84	91
IMP-69	88	88	87	86	87	84	84	90
IMP-20	88	88	87	85	86	84	84	89
IMP-23	88	88	88	85	87	84	84	90
IMP-39	85	85	85	83	85	83	83	89
IMP-33	83	83	83	83	84	83	83	89
IMP-17	82	82	81	81	83	82	82	89
IMP-13	82	82	81	81	83	82	82	89
IMP-37	82	81	81	81	84	82	82	88
IMP-84	83	82	82	80	83	82	82	88
IMP-90	83	83	83	82	83	82	82	83
IMP-12	83	83	82	81	83	82	82	84
IMP-63	83	83	83	82	84	83	83	83
IMP-48	91	91	90	91	81	80	80	84
IMP-32	91	91	91	91	81	80	80	84
IMP-14	91	91	91	91	81	80	80	84
IMP-65	91	91	90	91	82	81	81	83
IMP-54	91	91	90	91	81	80	80	84
IMP-91	91	91	90	91	81	80	80	83
IMP-86	91	91	90	90	82	81	81	84
IMP-87	91	91	91	91	82	81	81	84
IMP-83	99	99	98	89	80	79	79	83
IMP-18	99	99	99	90	80	79	79	83
IMP-56	99	99	98	89	80	79	79	83
IMP-49	100	99	99	89	80	79	79	83
IMP-71	99	100	99	89	80	79	79	82
IMP-75	99	99	100	88	79	78	78	82
IMP-46	89	89	88	100	81	80	80	81
IMP-35	80	80	79	81	100	97	97	80
IMP-31	79	79	78	80	97	100	99	80
IMP-92	79	79	78	80	97	99	100	80
IMP-95	83	82	82	81	80	80	80	100
IMP-27	81	81	80	81	80	79	79	96
IMP-64	81	81	80	81	80	79	79	96
IMP-67	81	80	80	81	80	79	79	96

Table S3. (continued)								
Identity (%)	IMP-27	IMP-64	IMP-67					
IMP-52	79	79	78					
IMP-42	79	80	79					
IMP-79	79	80	79					
IMP-70	80	80	79					
IMP-66	79	80	79					
IMP-60	79	80	79					
IMP-30	79	80	79					
IMP-34	79	80	79					
IMP-1	80	80	79					
IMP-61	79	80	79					
IMP-80	79	80	79					
IMP-78	80	80	79					
IMP-6	80	80	80					
IMP-3	80	80	79					
IMP-25	80	80	79					
IMP-76	79	80	79					
IMP-40	79	79	78					
IMP-77	79	80	79					
IMP-10	79	80	79					
IMP-88	79	79	78					
IMP-55	78	79	78					
IMP-38	80	81	80					
IMP-4	80	80	80					
IMP-59	80	80	79					
IMP-26	80	80	79					
IMP-89	79	80	79					
IMP-5	82	82	81					
IMP-85	81	82	81					
IMP-28	81	82	81					
IMP-94	81	81	80					
IMP-81	83	82	82					
IMP-51	82	81	81					
IMP-43	81	80	80					
IMP-7	81	81	81					
IMP-73	81	80	80					
IMP-15	83	83	83					
IMP-62	83	83	83					
IMP-29	80	81	80					
IMP-82	81	80	81					
IMP-9	80	80	80					
IMP-45	80	80	81					
IMP-53	80	80	80					
IMP-21	83	82	82					
IMP-11	83	83	83					
IMP-68	83	83	83					
IMP-41	83	82	82					
IMP-44	82	82	82					
IMP-22	83	83	83					
IMP-58	83	82	82					
IMP-16	83	83	83					

IMP-74	83	83	83					
IMP-93	83	82	82					
IMP-2	87	87	87					
IMP-19	87	87	87					
IMP-8	87	87	87					
IMP-24	87	87	87					
IMP-96	88	87	87					
IMP-69	88	87	87					
IMP-20	87	86	86					
IMP-23	87	87	87					
IMP-39	87	86	86					
IMP-33	86	86	86					
IMP-17	86	85	85					
IMP-13	85	85	85					
IMP-37	84	84	84					
IMP-84	85	84	84					
IMP-90	83	82	82					
IMP-12	83	83	83					
IMP-63	83	83	83					
IMP-48	84	84	84					
IMP-32	84	84	84					
IMP-14	84	84	84					
IMP-65	83	83	83					
IMP-54	84	84	84					
IMP-91	83	83	83					
IMP-86	84	84	84					
IMP-87	84	84	84					
IMP-83	82	82	81					
IMP-18	82	82	81					
IMP-56	82	82	82					
IMP-49	81	81	81					
IMP-71	81	81	80					
IMP-75	80	80	80					
IMP-46	81	81	81					
IMP-35	80	80	80					
IMP-31	79	79	79					
IMP-92	79	79	79					
IMP-95	96	96	96					
IMP-27	100	99	99					
IMP-64	99	100	99					
IMP-67	99	99	100					
